# Capture of MicroRNA–Bound mRNAs Identifies the Tumor Suppressor miR-34a as a Regulator of Growth Factor Signaling

**DOI:** 10.1371/journal.pgen.1002363

**Published:** 2011-11-10

**Authors:** Ashish Lal, Marshall P. Thomas, Gabriel Altschuler, Francisco Navarro, Elizabeth O'Day, Xiao Ling Li, Carla Concepcion, Yoon-Chi Han, Jerome Thiery, Danielle K. Rajani, Aaron Deutsch, Oliver Hofmann, Andrea Ventura, Winston Hide, Judy Lieberman

**Affiliations:** 1 Immune Disease Institute, Program in Cellular and Molecular Medicine, Children's Hospital Boston, Boston, Massachusetts, United States of America; 2 Department of Pediatrics, Harvard Medical School, Boston, Massachusetts, United States of America; 3 Genetics Branch, National Cancer Institute, National Institutes of Health, Bethesda, Maryland, United States of America; 4 Department of Biostatistics, Harvard School of Public Health, Boston, Massachusetts, United States of America; 5 Department of Cancer Biology and Genetics, Memorial Sloan Kettering Cancer Center, New York, New York, United States of America; University of California San Francisco, United States of America

## Abstract

A simple biochemical method to isolate mRNAs pulled down with a transfected, biotinylated microRNA was used to identify direct target genes of miR-34a, a tumor suppressor gene. The method reidentified most of the known miR-34a regulated genes expressed in K562 and HCT116 cancer cell lines. Transcripts for 982 genes were enriched in the pull-down with miR-34a in both cell lines. Despite this large number, validation experiments suggested that ∼90% of the genes identified in both cell lines can be directly regulated by miR-34a. Thus miR-34a is capable of regulating hundreds of genes. The transcripts pulled down with miR-34a were highly enriched for their roles in growth factor signaling and cell cycle progression. These genes form a dense network of interacting gene products that regulate multiple signal transduction pathways that orchestrate the proliferative response to external growth stimuli. Multiple candidate miR-34a–regulated genes participate in RAS-RAF-MAPK signaling. Ectopic miR-34a expression reduced basal ERK and AKT phosphorylation and enhanced sensitivity to serum growth factor withdrawal, while cells genetically deficient in miR-34a were less sensitive. Fourteen new direct targets of miR-34a were experimentally validated, including genes that participate in growth factor signaling (*ARAF* and *PIK3R2*) as well as genes that regulate cell cycle progression at various phases of the cell cycle (cyclins D3 and G2, *MCM2* and *MCM5*, *PLK1* and *SMAD4*). Thus miR-34a tempers the proliferative and pro-survival effect of growth factor stimulation by interfering with growth factor signal transduction and downstream pathways required for cell division.

## Author Summary

microRNAs (miRNAs) are small RNAs that regulate gene expression by binding to mRNAs bearing a partially complementary sequence. miRNAs decrease the stability or translation of mRNA targets, leading to reduced protein expression. Understanding the biological function of a miRNA requires identifying its targets. Here we developed a sensitive and specific biochemical method to identify candidate microRNA targets that are enriched by pull-down with a tagged, transfected microRNA mimic. The method was applied to miR-34a, a miRNA that inhibits cell proliferation. We found that miR-34a can potentially regulate hundreds of genes. Computational analysis of these genes suggested a novel function for miR-34a—suppression of the pro-proliferative response to diverse growth factors. This function complements the previously known role of miR-34a in blocking cell cycle progression. Thus, by reducing the expression of an extensive network of genes, miR-34a dampens growth factor signaling as well as its downstream consequences, promotion of cell survival and proliferation.

## Introduction

microRNAs (miRNAs) that promote cell differentiation, inhibit cell proliferation, or enhance DNA damage or stress-induced cell cycle arrest or death, and whose expression is reduced in some cancers, are candidate tumor suppressor genes [1]. One of the most well studied tumor suppressor miRNAs is miR-34a. Depending on cellular context [2], ectopic over-expression of miR-34a induces cell cycle arrest [3], senescence [4] or apoptosis [5]. miR-34a is up-regulated by p53 in response to DNA damage [6]–[8], but can also be transcriptionally activated independently of p53 [9], [10]. miR-34a is located on chromosome 1p36, a locus deleted in neuroblastoma, breast, thyroid, and cervical cancer [11], [12]. In other cancers, miR-34a expression is epigenetically reduced by hypermethylation [13]. miR-34a administration can inhibit tumor outgrowth in mice [4]. Thus miR-34a satisfies the criteria for a tumor suppressor gene.

The best way to understand the function of a miRNA is to identify the genes it regulates. In this study we sought to understand how miR-34a acts as a tumor suppressor by identifying its direct target genes. However, target gene identification is not straightforward because of the partial complementarity of the short ∼22 nt miRNA sequence with the miRNA recognition element (MRE) of the target gene [14]. MRE pairing to the miRNA seed region (nt 2–7) contributes significantly to target gene recognition and is the basis for the most successful target gene prediction algorithms [15], [16]. However, a perfect seed match is not necessary [17], [18] and does not guarantee targeting [19]. miRNA target prediction algorithms typically predict hundreds to thousands of putative miRNA target genes, but most predicted target genes are not bona fide targets and the best algorithms sometimes miss key targets [17], [19]–[21]. It is unclear how many target genes are in fact regulated by a given miRNA in any physiological context. Analysis of genes whose mRNA or protein expression decreases when a miRNA is overexpressed or increases when it is antagonized identifies genes that may be either direct targets or indirectly regulated [22]. Biochemical methods to capture RNA-induced silencing complex (RISC)-bound mRNAs potentially provide a more direct way to identify miRNA-regulated target genes [23]–[25]. However, immunoprecipitation has mostly been used to define the general features of miRNA-regulated mRNAs and their MREs, rather than to identify the targets of a particular miRNA.

Already 36 putative miR-34a targets have been validated by luciferase reporter assays. These targets strongly support miR-34a's role as a tumor suppressor. They include genes that promote cell cycle progression through the G_1_/S transition (*CCND1*, *CCNE2*, *CDK4*, *CDK6*, *MYC*, *MYCN* and *E2F3*) [3], [8], [10], [12], [26], enhance transcription (*MYB*, *HNF4A* and *FOXP1*) [9], [27], [28] or growth factor signaling (*MET*, *MEK1*, *AXL* and *RRAS*) [8], [29]–[32], inhibit apoptosis (*BCL2*) [33] or p53 activity (*YY1*, *MTA2*, *SIRT1* and *MAGE-A*) [5], [32], [34], [35], and promote stem cell survival (*NOTCH1*, *NOTCH2*, *LEF1 WNT1*, *DLL1*, *JAG1* and *CD44*) [32], [36]–[40]. The diversity of direct miR-34a targets suggests that miR-34a acts pleiotropically by regulating many genes.

To identify additional direct target genes of miR-34a without bias and understand better how miR-34a functions, we optimized a simple biochemical method to isolate mRNAs that bind to transfected biotinylated (Bi-)miR-34a [41], [42]. mRNAs significantly enriched in the Bi-miRNA pull-down with streptavidin relative to their cellular expression were candidate targets. The pull-down was performed in two unrelated cancer cell lines, K562 erythroleukemia cells and HCT116 colon carcinoma cells. p53 activates transcription of miR-34a [8]. Under basal conditions, p53-sufficient HCT116 cells highly express miR-34a, while p53-null K562 cells do not express it above background (data not shown). We selected disparate cell lines to identify genes that may be regulated in multiple cell types or more specifically in a particular context. Several thousand genes were significantly enriched in the miR-34a pull-down in each cell line and 982 were significantly enriched in both cell lines. Most known miR-34a target mRNAs expressed in these cells were pulled down with miR-34a. Despite the large number of genes significantly enriched in the miR-34a pull-down, 91% of a random list of 11 genes enriched in both cell lines contained miR-34a-regulated 3′UTR sequences. These results suggest that the pull-down is quite specific and that miR-34a potentially directly regulates hundreds of genes. Bioinformatic analysis of the pulled down genes or of genes down-regulated after miR-34a transfection suggested that miR-34a regulates a dense network of genes that transduce proliferative signals arising from growth factor stimulation. Multiple candidate target genes participate in RAS-RAF-MAPK signaling. In fact miR-34a knockout reduced sensitivity to growth factor withdrawal by serum starvation, while miR-34a transfection led to increased vulnerability. Fourteen novel miR-34a targets identified by the pull-down in both cell lines were experimentally verified, including *ARAF* and *PIK3R2* in the RAS-RAF-MAPK pathway, and additional target genes required for cell cycle progression, including cyclins D3 and G2, *MAD2L2*, *MCM2*, *MCM5* and *PLK1*.

## Results

### Isolation of mRNAs bound to a transfected biotinylated–miRNA

**Figure 1:**
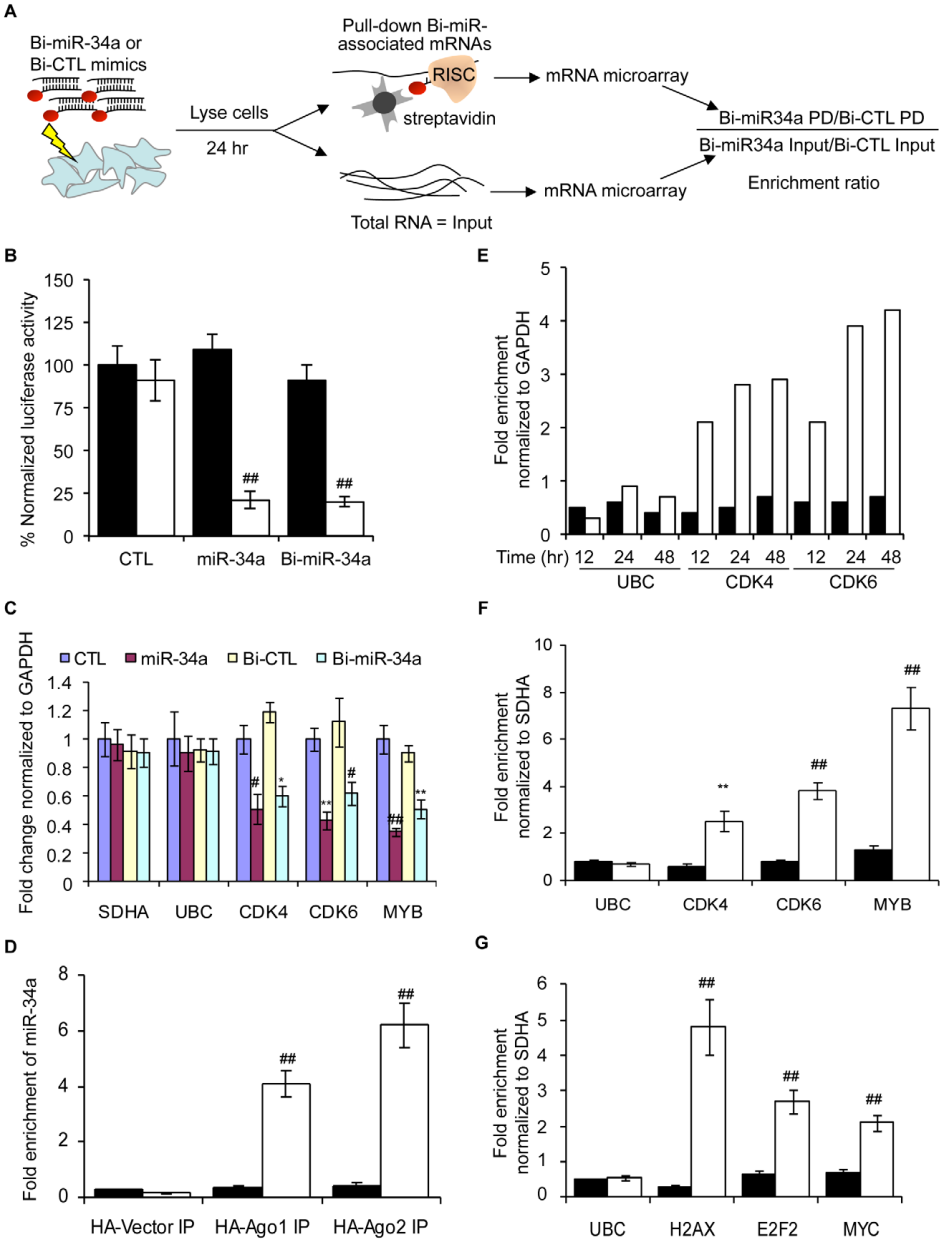
The Biotin-miRNA pulldown method. (A) Schematic of the Bi-miRNA pull-down (PD) assay. (B) Activity of 3′-biotinylated miR-34a (Bi-miR-34a) is similar to unbiotinylated miR-34a mimics by dual luciferase assay performed in HeLa cells cotransfected with psiCHECK-2 vector (black) or psi-CHECK-2 bearing a perfectly complementary sequence to miR-34a (psiCHECK-2-AS-miR-34a, white). Transfection with cel-miR-67 is the control (CTL). Luciferase expression was assayed after 24 hr; results are normalized to cells transfected with the luciferase vector and the CTL miRNA. (C) Bi-miR-34a efficiently silences known miR-34a targets *CDK4*, *CDK6* and *MYB*. K562 cells were transfected with CTL miRNA, miR-34a, Bi-CTL or Bi-miR-34a mimics for 48 hr. Expression was measured by qRT-PCR normalized to *GAPDH*. The housekeeping genes *SDHA* and *UBC* are negative controls. (D) Cytoplasmic lysates from K562 cells were prepared 48 hr after cotransfection with Bi-CTL (black) miRNA or Bi-miR-34a (white) and a plasmid encoding HA-Ago1, HA-Ago2, or empty vector. Enrichment of miR-34a by HA immunoprecipitation was measured by qRT-PCR normalized to *U6*. Enrichment of Bi-miR-34a in the HA-immunoprecipitates suggests that Bi-miR-34a is incorporated into RISC. (E) Bi-miR-34a pull-downs optimally enrich targets 24 or 48 hr after transfection. K562 cells were transfected in duplicate with Bi-CTL (black) or Bi-miR-34a (white) mimics for the indicated times. Enrichment of known miR-34a targets (*CDK4* and *CDK6*) or control genes (*GAPDH* and *UBC*) was assessed by qRT-PCR relative to *GAPDH*. (F) The streptavidin pull-down enriches for miR-34a target genes in K562 cells transfected with Bi-CTL (black) or Bi-miR-34a (white) mimics. (G) Known miR-24 target mRNAs (*H2AX*, *E2F2* and *MYC*) are also pulled down with Bi-miR-24 in HepG2 cells reverse transfected 48 hr earlier with Bi-CTL (black) or Bi-miR-24 (white). Enrichment of target mRNAs in (F) and (G) was analyzed by qRT-PCR relative to *SDHA*. In all panels, data represent mean ± SD of 3 independent experiments. *, p<0.05, #, p<0.01, **, p<0.005, ##, p<0.001.

We modified a method [5] for capturing miRNA-mRNA complexes using streptavidin-coated beads from cells transfected with miR-34a biotinylated at the 3′-end of the mature strand. Control samples were transfected with a biotinylated *C. elegans* miRNA (Bi-cel-miR-67) (Figure 1A). Biotinylation did not interfere with miRNA-mediated gene suppression as measured by luciferase reporter assay (Figure 1B). Over-expressing Bi-miR-34a or miR-34a in K562 cells also similarly suppressed expression of known miR-34a target genes (Figure 1C). Moreover, immunoprecipitation of HA-tagged Ago1 or Ago2 in K562 cells cotransfected with Bi-miR-34a specifically enriched for miR-34a by ∼4-fold and ∼6-fold, respectively (Figure 1D). Thus the Bi-miRNA is incorporated into the RISC and functions like the unbiotinylated miRNA.

We next optimized conditions to capture known target gene mRNAs. In the Bi-miR-34a pull-down of K562 cells, known miR-34a target transcripts *CDK4* and *CDK6*, but not UBC (a housekeeping gene), were enriched 12 hr after transfection, and their capture plateaued at 24–48 hr (Figure 1E). Therefore, 24 hr was chosen for subsequent experiments. The specificity of the pull-down and applicability to other cell types was verified since *CDK4*, *CDK6* and *MYB* mRNAs were consistently enriched by transfection of Bi-miR-34a, but not Bi-cel-miR-67, in K562 (Figure 1F) and HCT116 (Figure S1A) cells. Streptavidin beads did not enrich for non-target *SDHA* and *UBC* mRNAs, and the specific target mRNAs were not pulled down in cells transfected with unbiotinylated miR-34a (data not shown). miR-34a was specifically enriched >40-fold in the Bi-miR-34a pull-down compared to the input lysate (Figure S1B). Modifications of the pull-down to include formaldehyde cross-linking and/or pre-isolation of RNAs in high molecular weight cellular fractions reduced the amount of captured RNA, but did not improve the relative enrichment for known target gene mRNAs (data not shown). To confirm that association of Bi-miRNAs with target mRNAs was not a post-lysis artifact, we performed streptavidin pull-downs after adding Bi-miR-34a or Bi-cel-miR-67 to cytoplasmic extracts of untransfected K562 cells. *CDK4*, *CDK6* and *MYB* mRNAs were not enriched when Bi-miR-34a was added post-lysis (Figure S1C). The general applicability of the pull-downs to enrich for miRNA target genes was also verified for another miRNA, miR-24 in HepG2 cells. Bi-miR-24 capture enriched for 3 known miR-24 targets (*H2AFX*, *E2F2* and *MYC* [43]) by 2–5-fold (Figure 1G).

### Sensitivity of the Bi-miR-34a pull-down

We next used gene expression microarrays to identify putative miR-34a targets captured by Bi-miR-34a in duplicate experiments from K562 (p53 deficient) and HCT116 cells (p53 proficient) (Table S1). mRNA abundance in the streptavidin pull-down and input in Bi-miR-34a-transfected cells were separately normalized to their levels in Bi-cel-miR-67-transfected cells. For each biological replicate, the ratio of the abundance of the pull-down mRNA compared to the input mRNA for cells transfected with Bi-miR-34a versus Bi-cel-miR-67 was calculated, averaged and used to define the enrichment ratio {Bi-miR-34a PD/Bi-cel-miR-67 PD}/{Bi-miR-34a input/Bi-cel-miR-67 input}. Normalizing to the input improved identification of true targets in 2 ways – by reducing the background caused by highly abundant mRNAs that associate with streptavidin beads nonspecifically and by incorporating a measure of mRNA knockdown into the denominator of the ratio.

**Figure 2:**
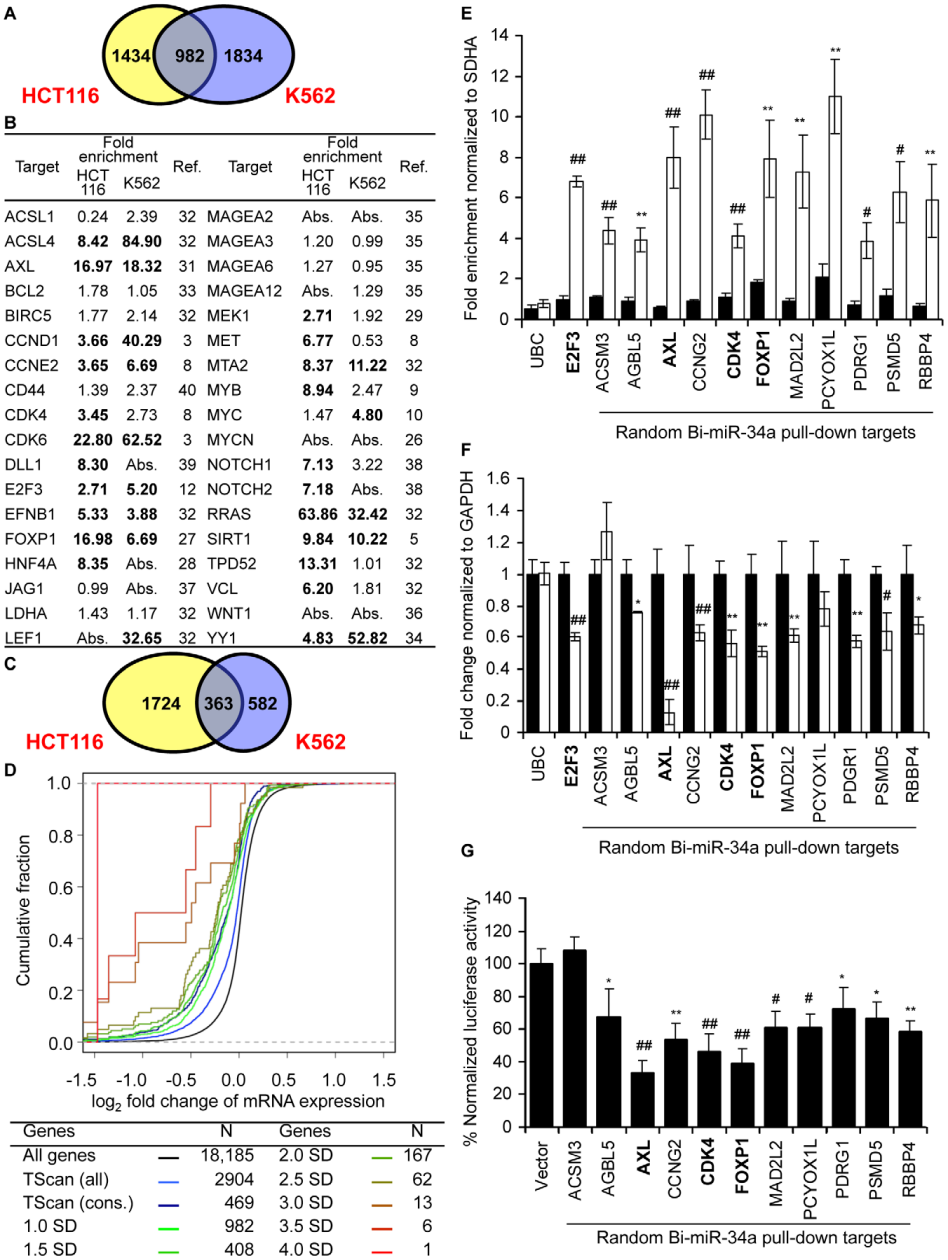
miR-34a pulls down transcripts of known and novel direct targets of miR-34a. (A) Overlap of genes enriched ≥1 SD in gene expression microarray analysis of Bi-miR-34a pull-downs from HCT116 and K562 cells. (B) Enrichment of previously described miR-34a target gene mRNAs in Bi-miR-34a pull-downs from HCT116 and K562 cells. Genes enriched ≥1 SD are indicated in bold. Abs = not expressed. (C) Genes down-regulated by ≥20% after miR-34a over-expression. (D) mRNA expression of candidate miR-34a targets identified by Bi-miR-34a pull-down in both HCT116 and K562 cells decreases after miR-34a over-expression. Cumulative distribution plots compare the extent of mRNA reduction of genes enriched to varying degrees in the Bi-miR-34a pull-down with conserved (cons) or all TargetScan (TScan)-predicted targets. Genes whose mRNAs are more highly enriched in the pull-down are progressively more likely to have reduced expression. (E) Pull-down of 11 mRNAs randomly selected from the set of genes enriched ≥2.5-fold by microarray in both HCT116 and K562 Bi-miR-34a pull-downs is confirmed by qRT-PCR relative to *SDHA* in K562 cells transfected for 24 hr with Bi-CTL (black) or Bi-miR-34a (white). *UBC* is a negative control and *E2F3* is a positive control. Three previously validated miR-34a targets (*AXL*, *CDK4* and *FOXP1*) in the random list of genes are indicated in bold. (F) miR-34a over-expression significantly decreases the expression of 9 of 11 of the randomly chosen candidate target genes. K562 cells were transfected with CTL miRNA (black) or miR-34a (white) mimics for 72 hr. Expression of random targets was measured by qRT-PCR normalized to *GAPDH*. Expression of 2 randomly selected genes (*ACSM3* and *PCYOX1L*) and the housekeeping mRNA *UBC* didn't change significantly. (G) miR-34a targets the 3′UTR of 10 of 11 randomly chosen targets. Luciferase activity was measured 48 hr after HeLa cells were cotransfected with the luciferase reporter psiCHECK2 bearing the 3′UTR of each gene and CTL miRNA or miR-34a mimics. Results obtained after miR-34a transfection were normalized to CTL miRNA. In (E–G), data represent mean ± SD of 3 independent experiments. *, p<0.05, #, p<0.01, **, p<0.005, ##, p<0.001.

The miR-34a pull-downs enriched for 2416 genes in HCT116 cells (by ≥1 standard deviation (SD), enrichment ratio ≥2.5) and for 2816 genes in K562 cells (≥1 SD, enrichment ratio ≥3.3) (Figure 2A). The overlap of genes enriched ≥1 SD in both of these unrelated cell lines was 982 genes. To determine the sensitivity of the pull-down, we first looked at how many of the 36 published targets of miR-34a were captured in the K562 or HCT116 pull-downs (Figure 2B). Of the known expressed targets, 22 of 31 mRNAs (71%) were enriched in HCT116 cells and 14 of 29 (48%) were enriched in K562 cells. It should be noted that the choice of cut-off is somewhat arbitrary. Two additional known targets had enrichment ratios of 2.5–3.2 in K562 cells. The enrichment ratio ranged from 2.7–85. 12 genes were identified in both pull-downs. The enrichment ratio for the shared hits was not significantly different in K562 cells, which do not express miR-34a, compared to HCT116 cells, which do, suggesting that the pull-downs efficiently captured miR-34a targets even in cells that express endogenous miR-34a.

### Analysis of genes down-regulated by miR-34a over-expression

To compare the mRNAs that associate with miR-34a to mRNAs that decrease with miR-34a over-expression, we measured mRNA abundance in cells transfected with miR-34a or cel-miR-67 by gene expression microarrays (Table S1). Genes whose mean mRNA level ratio decreased by at least 20% after miR-34a transfection were considered to be down-regulated either directly or indirectly by miR-34a. With this arbitrary cut-off (∼1 SD), 2087 genes were down-regulated in HCT116 cells and 945 genes were down-regulated in K562 cells (Figure 2C). About a third of these transcripts in both cell lines were also pulled down with Bi-miR-34a (30% in HCT116, 36% in K562).

### Down-regulated and pulled down mRNAs are enriched for miR-34a seed sequences

Many miRNA targets contain a perfect match to the miRNA seed region in their 3′UTR. We examined the frequency of 3′UTR matches to all hexamer sequences in miR-34a in the pull-down and down-regulated gene sets relative to all genes probed on the microarray (Figure S2A). Hexamer matches to nt 2–7 in the miR-34a seed region were significantly enriched in the pull-down (HCT116 p = 1.8E-95; K562 p = 2.4E-11) and down-regulated (HCT116 p = 1.7E-24; K562 p = 1.0E-11) datasets. There was also significant enrichment in the HCT116 pull-down genes for nt 13–19 exact matches, suggesting that base-pairing there enhances miRNA binding, as has previously been shown [44]. In both cell lines, seed enrichment was greater for the overlapping set of genes that was both pulled down and down-regulated by miR-34a. For genes in this overlap, exact matches to nt 2–7 were 1.8–2.0-fold more frequent per kb of 3′UTR than for all genes on the microarray. These data suggest that genes in the overlap may be more likely to be direct targets than genes identified by only one method or that a perfect seed match might enhance miRNA-mediated mRNA decay.

We next examined hexamer enrichment in the 982 genes enriched ≥1 SD in pull-downs from both HCT116 and K562 cells (Figure S2B). Seed matches were most enriched in the 3′UTRs of these genes, with the nt 2–7 match being the most abundant (1.7 fold more abundant than in all genes on the microarray (p = 8.4E-39). The coding region (CDS) of these genes also contained a highly significant enrichment for hexamer seed matches (p = 6.1E-13). These results are consistent with recent cross-linked RISC pull-downs that suggest that 25–50% of MREs may be in the CDS [23], [25]. There was also a modest enrichment of hexamers matching the seed in the 5′UTR (p = 0.005). Thus the pull-down and down-regulated mRNAs were enriched for expected miRNA target sequence features.

We next analyzed whether mRNA expression of the enriched genes was reduced by miR-34a transfection in HCT116 cells (Figure 2D). The mRNAs of the 982 genes enriched in the miR-34a pull-down by ≥1 SD in both cell lines were significantly down-regulated after miR-34a transfection compared to the set of all genes expressed in the cell (p = 4.7E-80). The extent of down-regulation was comparable to the set of 469 TargetScan-predicted, evolutionarily conserved targets of miR-34a and significantly greater than in the larger list of 2904 poorly conserved, TargetScan-predicted genes (p = 1.6E-20). Increasing the cutoff for the enrichment ratio in the pull-down led to a greater proportion of highly down-regulated genes, indicating that a higher enrichment ratio correlates with more effective mRNA degradation and/or that highly enriched mRNAs are more likely to be miR-34a targets. Thus, the Bi-miR-34a pull-down enriches for known sequence and gene expression characteristics of *bona fide* miRNA targets.

### Genes enriched in the miR-34a pull-down of both cell lines have a high probability of being direct miR-34a targets

To determine the specificity of the pull-down, we generated a random list (Table S2) of 11 genes enriched >2.5 fold in both pull-downs (median enrichment 3.5-fold, range 2.5–17.3). The random list contained 3 known target genes (*AXL*, *CDK4* and *FOXP1*; *AXL* and *FOXP1* were not known when the list was generated). First, qRT-PCR analysis verified that the random gene mRNAs are pulled down by Bi-miR-34a and not Bi-cel-miR-67. All 11 mRNAs were enriched (∼4–10 fold) by Bi-miR-34a pull-down in K562 cells, validating the microarray results (Figure 2E). miR-34a over-expression significantly down-regulated mRNA levels of 9 of 11 genes by 25–90% (Figure 2F). *PCYOX1L* expression declined by 20%, but the change was not significant. To test whether the 3′UTR of each gene could be regulated by miR-34a, the full 3′ UTR of each gene was cloned into a dual luciferase reporter plasmid. miR-34a repressed the 3′UTRs of 10 of 11 genes by ∼20–80% (Figure 2G). Thus, miR-34a could regulate the 3′UTR of 91% of a random set of genes enriched in both miR-34a pull-downs. These results suggest that the Bi-miRNA pull-down is highly specific for identifying direct miRNA targets. An important implication of the large number of genes in the overlapping target list and the low false positive rate is that miR-34a is capable of regulating hundreds of genes.

### miR-34a directly regulates growth factor signaling and cell cycle progression

**Figure 3:**
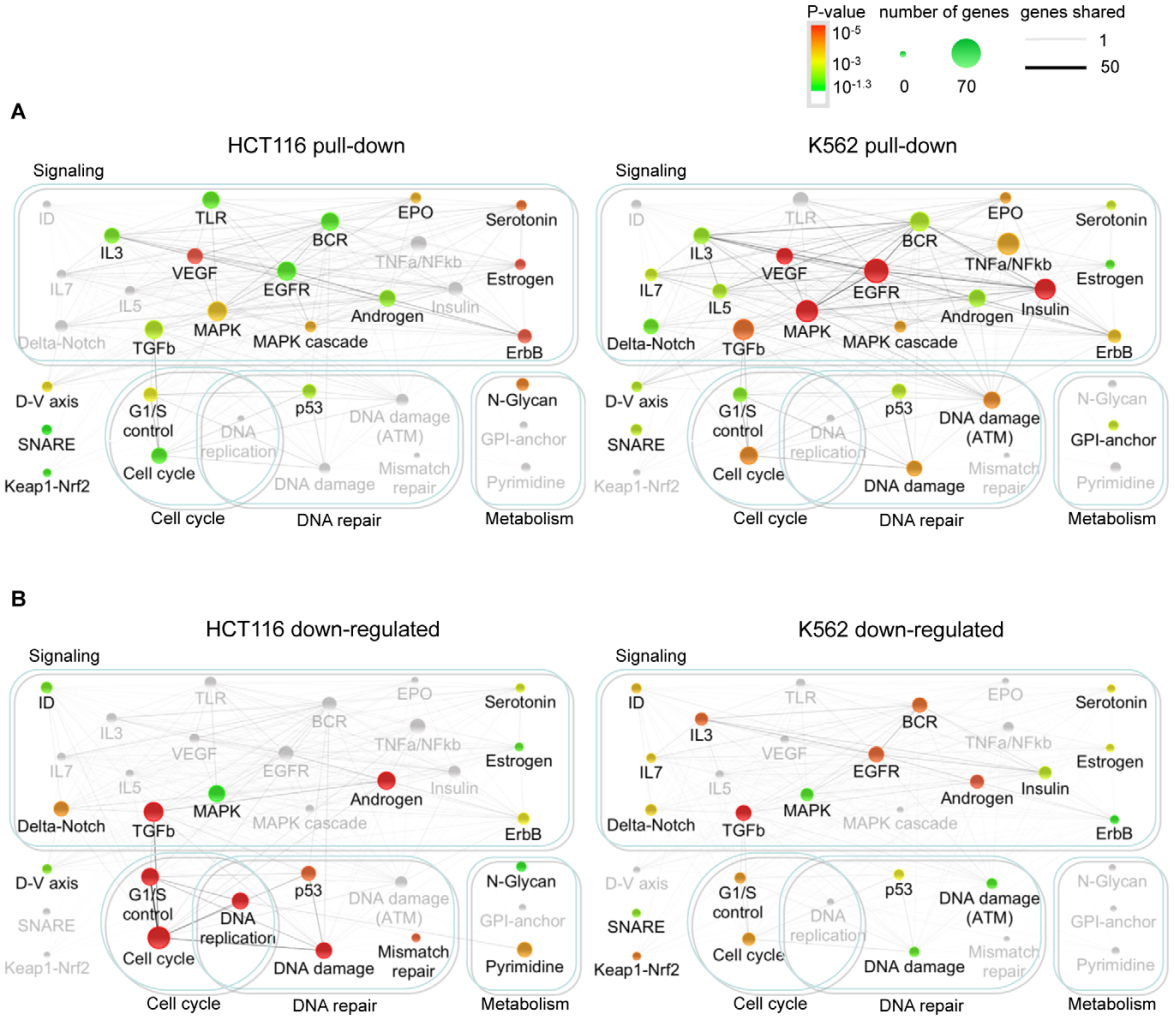
Genes in the Bi-miR-34a pull-down or down-regulated by miR-34a over-expression are enriched in growth factor signaling, cell cycle progression, and DNA repair pathways. Network of canonical pathways (Wikipathways and KEGG) significantly enriched for genes identified by Bi-miR-34a pull-down (A) or down-regulated following miR-34a over-expression (B) in HCT116 and K562 cells. Each pathway is represented by a node in the network. The node size increases with the number of identified genes in the pathway and the node color represents the p-value based on the hypergeometric distribution (see key). Pathways that are not significantly enriched in an experiment are still shown, but are in gray. The number of genes shared between two pathways is represented by an edge whose thickness increases with the number of shared genes. The pull-down enriched pathways (A) suggest that miR-34a extensively targets growth factor, signal transduction and cell cycle control pathways. The integrated outcome of both direct and indirect effects of miR-34a on gene expression in (B) is suppression of expression of genes participating in downstream signaling, cell cycle and DNA repair pathways.

To understand miR-34a's biological functions, we analyzed the cellular pathways whose genes were most enriched in the Bi-miR-34a pull-downs (Figure 3A). In both K562 and HCT116 cells, Bi-miR-34a pull-downs enriched for genes in pathways related to growth factor signaling and cell cycle control. Bi-miR-34a pull-downs enriched significantly for genes in the EGFR, TGF-β, interleukin, estrogen, and androgen receptor signaling pathways (Figure 3A). Many of these pathways utilize common downstream signaling molecules and have a well-established link to cancer. Genes in the MAPK pathway, activated by most growth factors, were highly enriched in the pull-downs for both cell lines. Growth factor signaling also activates cell proliferation. Genes involved in cell cycle regulation, especially the G_1_/S transition, and the p53 response were enriched in both pull-downs, consistent with previously described targets and roles of miR-34a [3], [4], [7], [8].

We performed a similar pathway enrichment analysis for genes down-regulated by miR-34a (Figure 3B), which includes both direct and indirect miR-34a targets. The downstream effects of growth factor signaling on cell proliferation and p53 activation were more prominent in the down-regulated genes than in the pulled-down gene set, especially in p53-sufficient HCT116 cells. Cell cycle and DNA repair pathways were enriched in genes down-regulated by miR-34a in both K562 and HCT116 cells. These results suggest that miR-34a directly inhibits growth factor signal transduction and cell cycle progression pathways, culminating in reduced expression of genes needed for cell proliferation.

A pathway enrichment analysis of the TargetScan-predicted targets of miR-34a (Figure S3) also highlighted the most significantly enriched pathways in the experimental pull-down and down-regulated gene sets, notably TGFβ and MAPK signaling and cell cycle and G_1_/S transition. However, the significance of the enrichment was weaker and the strong role of miR-34a in growth factor signaling was less obvious.

### miR-34a regulates a dense network of genes involved in signal transduction and cell cycle progression

**Figure 4:**
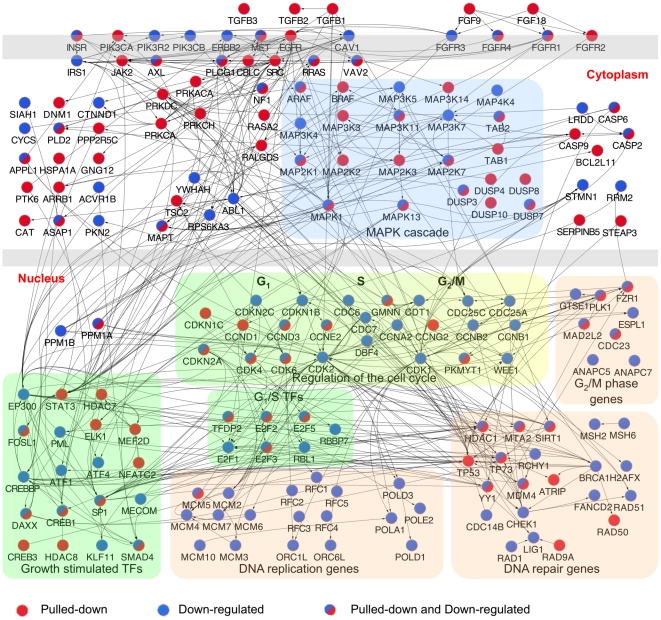
Interactome of genes in the enriched canonical pathways pulled down with Bi-miR-34a and/or down-regulated by miR-34a over-expression. Interactome of products of genes identified by Bi-miR-34a pull-down (red) or down-regulated by miR-34a over-expression (blue) in significantly enriched pathways (Figure 3) in HCT116 cells. Edges represent physical interactions. A dense network of genes involved in growth factor signaling and downstream effects on cell cycle progression and DNA repair is implicated.

To begin to understand regulation of growth factor signaling and cell proliferation at the gene level by miR-34a, an interactome of pulled down or down-regulated genes in HCT116 cells that participate in the significantly enriched pathways was generated (Figure 4). miR-34a potentially regulates the expression of critical genes involved in virtually every step and branch of growth factor signal transduction from ligand binding to downstream growth-promoting transcription factors. The putative direct targets included genes encoding multiple TGFβ and FGF isoforms, receptors for EGF, FGF, and insulin, and several oncogenic receptor tyrosine kinases, including *MET* and *AXL*. Several genes operating proximally in signal transduction, including *SRC*, *PLCG1* and *VAV2*, were selectively pulled down. miR-34a targets also included protein kinase subunits that activate downstream signaling, including subunits of protein kinase A and C. In the RAS-RAF-MAPK signal transduction pathway, putative directly regulated genes included *RRAS* and *RASA2*, *ARAF* and *BRAF*, *JAK2*, and 11 *MAPK* genes. Although knockdown of most of the targets would be expected to inhibit cellular activation by diverse growth factors, the genes also encode for some important inhibitors, including the ubiquitin ligase *CBLC*, *RASA2*, and 5 *DUSP* genes (MAPK phosphatases). The pull-down also captured 76 transcripts of transcription factors, including some that orchestrate the transcriptional response to signal transduction (including *STAT3*, *CREB1* and *CREB3*, *SP1*, *ELK1* and *SMAD4*).

A major downstream effect of growth factor signaling and its activated transcription factors is to stimulate cell proliferation. miR-34a is already known to suppress *E2F3* and some key cyclins and cyclin-dependent kinases that regulate the G_1_/S transition. The miR-34a pull-down enriched for additional cyclins (*CCND3*, *CCNG2*), but also for transcripts of genes that inhibit the kinases that promote exit from G_1_ (*CDKN1C* that encodes p57(KIP2), *CDKN2A* (p14(ARF)). Other enriched transcripts include *MCM5*, whose product is required to initiate DNA replication, and several genes required for mitosis (*PLK1*, *MAD2L2* and *CDC23*). Ectopic miR-34a expression led to down-regulation of mRNAs for many genes needed to replicate DNA, including 2 members of the initiating complex that assembles at origins of DNA replication, 7 components of the MCM complex, 4 DNA polymerases, and 5 components of the RFC complex, a cofactor for DNA polymerase. These results suggest that miR-34a not only interferes with the signaling that transduces the growth factor response, but also directly and indirectly suppresses the expression of numerous genes needed for cell proliferation.

### miR-34a regulates cellular responses to growth factor signaling

**Figure 5:**
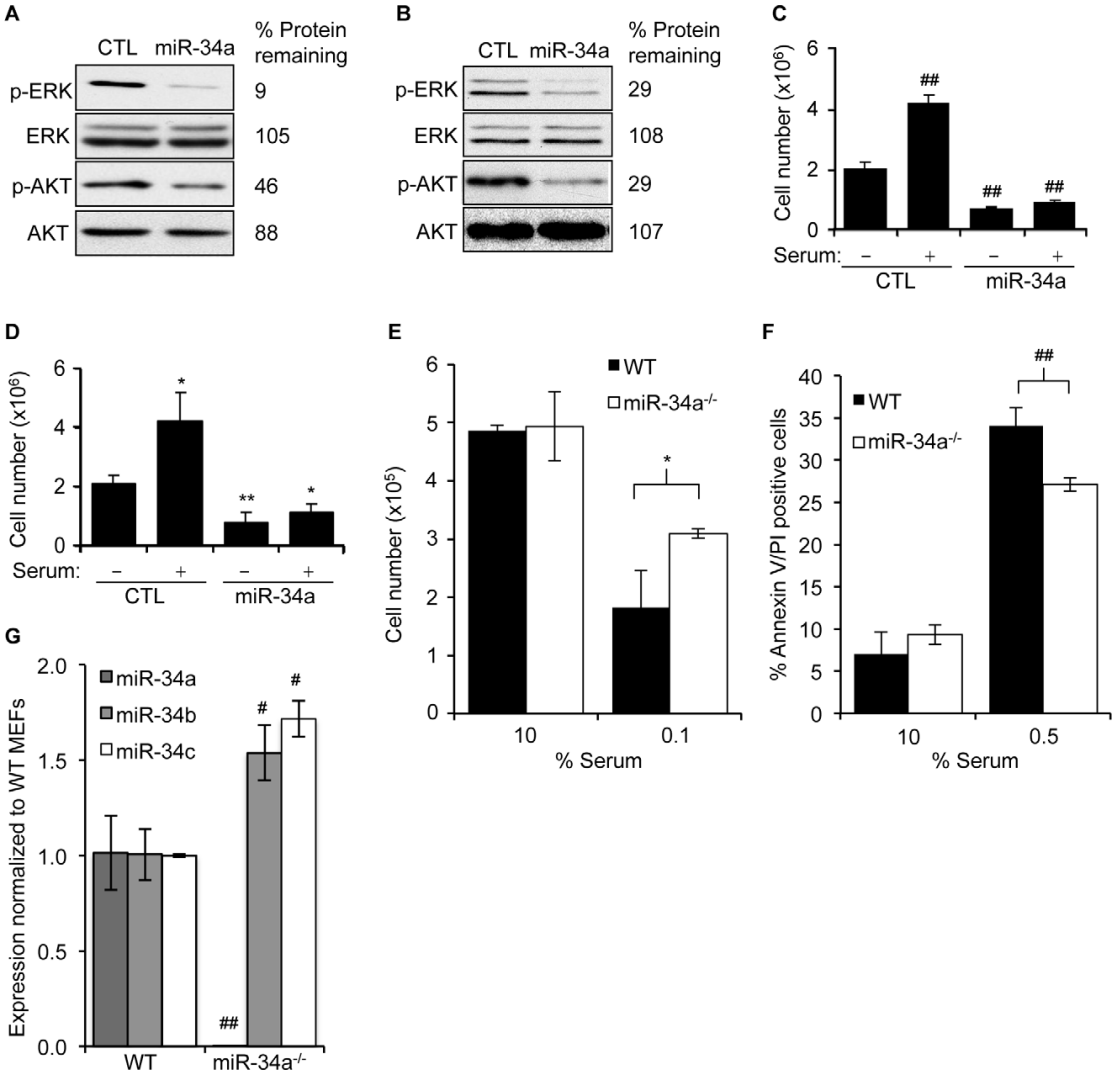
miR-34a expression suppresses cellular activation in response to serum growth factors. (A,B) miR-34a over-expression reduces basal phosphorylation of AKT and ERK as measured by immunoblot 48 hr after transfection of HCT116 (A) and HeLa (B) cells with control (CTL) miRNA or miR-34a mimics. Abundance of total ERK and AKT doesn't change. (C,D) miR-34a over-expression reduces cell proliferation in the absence of serum and suppresses the proliferative response of HCT116 (C) and HeLa (D) cells 24 hr after adding serum. (E) Total numbers of miR-34a^+/+^ or miR-34a^−/−^ MEFs after 24 hr of culture in 10% serum (10%) or 24 hr in 10% serum followed by 24 hr in 0.1% serum (0.1%). MEFs sufficient for miR-34a were more sensitive to serum starvation. (F) miR-34a^+/+^ MEFs were more prone to apoptosis than miR-34a^−/−^ MEFs after 24 hr of culture in reduced serum. (G) Expression of miR-34 family members in miR-34^+/+^ (WT) and miR-34^−/−^ MEFs assessed by qRT-PCR. miR-34a^−/−^ MEFs expressed higher levels of miR-34b and miR-34c. In C,D,F, and G, data represent mean ± SD of 3 independent experiments. *, p<0.05, #, p<0.01, **, p<0.005, ##, p<0.001.

The Ras–extracellular signal-regulated kinase (ERK) and phosphoinositide 3-kinase (PI3K)–AKT pathways are key transducers of the cellular response to growth factors. Since many candidate miR-34a target gene products act in pathways converging on ERK and AKT activation, we analyzed the effect of miR-34a over-expression on ERK and AKT phosphorylation. miR-34a transfection reduced basal phosphorylation of ERK and AKT in HCT116 and HeLa cells (Figure 5A, 5B), but not in A549 cells (Figure S4A). miR-34a over-expression both reduced basal proliferation in the absence of serum and blunted the ability of HCT116 (Figure 5C), HeLa (Figure 5D) and A549 (Figure S4B) cells to proliferate in response to serum growth factors. Conversely, immortalized mouse embryonic fibroblasts (MEFs) genetically deficient in miR-34a were more resistant to serum starvation than WT MEFs (Figure 5E). Apoptosis measured by annexin V and propidium iodide staining was also significantly reduced in miR-34a^−/−^ MEFs compared to wild-type MEFs after 24 hours of serum starvation (Figure 5F). Despite the strong difference in cell survival in cells deficient in miR-34a, expression of several known miR-34a targets did not differ significantly between wild-type and miR-34a^−/−^ MEFs (data not shown). The lack of a notable difference may be due in part to compensatory up-regulation of miR-34b and miR-34c in miR-34a^−/−^ MEFs (Figure 5G). These data suggest that miR-34a dampens the basal state of activation of proliferative and pro-survival pathways mediated by AKT and ERK by down-modulating multiple genes whose products contribute to their phosphorylation.

### miR-34a directly targets genes that regulate ERK and AKT phosphorylation

To determine whether some of the candidate miR-34a target genes identified in the pull-down that participate in growth factor signaling are bona fide targets, we next tested miR-34a targeting of selected receptor-proximal (*AXL*, *MET* and *PIK3R2*) and more downstream (*ARAF* and *MEK1*) components of ERK and AKT signal transduction pathways. These 5 genes were both pulled down with Bi-miR-34a and down-regulated by miR-34a in HCT116 cells. ARAF is a serine/threonine protein kinase that phosphorylates and activates MEK1, which in turn phosphorylates ERK [45]. AXL is a receptor tyrosine kinase that stimulates cell proliferation and also promotes metastasis [46], [47]. PIK3R2 is a regulatory subunit of PI3K [48] and MET is a tyrosine kinase receptor that activates both PI3K and RAS [49]. *AXL*, *MET* and *MEK1* are described miR-34a targets [8], [29], [31], although *AXL* and *MEK1* were not known when these studies were performed.

**Figure 6:**
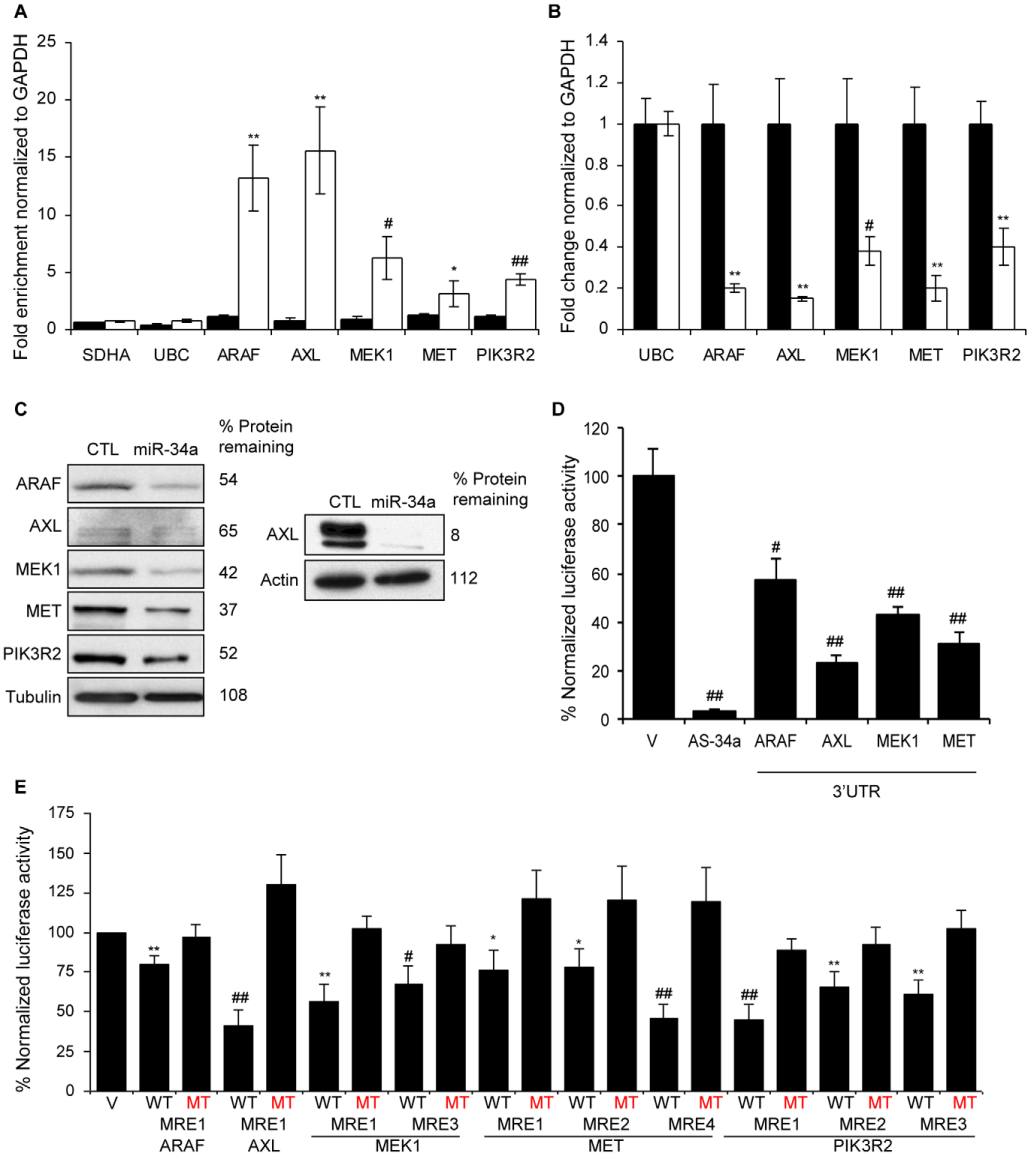
miR-34a directly inhibits growth factor signaling and signal transduction pathways by regulating novel genes. (A) Five genes involved in growth factor signaling or signal transduction are pulled down by Bi-miR-34a. HCT116 cells were transfected with Bi-cel-miR67 (CTL miRNA, black) or Bi-miR-34a (white) mimics for 24 hr and mRNA capture was measured by qRT-PCR normalized to *GAPDH*. Five of 5 (*ARAF*, *AXL*, *MEK1*, *MET* and *PIK3R2*) genes identified by microarrays, but not housekeeping mRNAs *SDHA* and *UBC*, are significantly enriched in the Bi-miR-34a pull-down. (B) miR-34a decreases *ARAF*, *AXL*, *MEK1*, *MET* and *PIK3R2* mRNAs, measured by qRT-PCR relative to *GAPDH*, in HCT116 cells transfected with CTL (black) or miR-34a (white) for 48 hr. *UBC* is a negative control gene. Relative mRNA levels were normalized to levels in CTL miRNA-transfected cells. (C) ARAF, AXL, MEK1, MET and PIK3R2 protein levels decline by immunoblot after miR-34a over-expression in HCT116 cells harvested 48 hrs after transfection with CTL miRNA or miR-34a mimic. Because of the low signal for AXL, an additional experiment probed for AXL with a longer exposure is shown at right. (D) miR-34a significantly regulates the 3′UTR of *ARAF*, *AXL*, *MEK1* and *MET* in HeLa cells co-transfected with a dual luciferase reporter bearing the 3′UTR of each gene and CTL miRNA or miR-34a for 48 hr. Insertion of a sequence fully complementary to miR-34a into the *Renilla luciferase* 3′UTR (AS-34a) is the positive control. Luciferase activity was normalized to results obtained with the empty vector (V). (E) Luciferase reporters bearing PITA-predicted wild-type (WT) MREs from each target gene are significantly repressed in HeLa cells cotransfected with miR-34a. Point mutations (MT) that disrupt base pairing with miR-34a rescue reporter expression. MRE sequences are provided in Figure S4.

The transcripts of all 5 genes were enriched 3–15-fold in the Bi-miR-34a pull-down by qRT-PCR, validating the microarray results (Figure 6A). Furthermore, over-expression of miR-34a down-regulated both the mRNA and protein levels of all 5 genes (Figure 6B, 6C). All but *ARAF* are also predicted miR-34a targets by TargetScan. To determine whether these genes are direct miR-34a targets, we tested the 3′UTRs for 4 of the genes (*ARAF*, *AXL*, *MEK1* and *MET*) by luciferase assay. miR-34a reduced reporter activity of these 3′UTRs by ∼40–75% (Figure 6D). Using the PITA algorithm [50] to identify potential MREs in their 3′UTRs, we found 1 potential MRE in *AXL*, 2 in ARAF, 3 in *MEK1*, 4 in *PIK3R2* and 5 in *MET* (Figure S4). We tested repression of these MREs by miR-34a using luciferase assays. All 5 genes contained at least one miR-34a-responsive MRE (Figure 6E). Point mutations that disrupt the MRE-miR-34a interaction restored luciferase activity, validating their regulation by miR-34a. Therefore, these 5 important genes in PI3K and MAPK signaling are all directly regulated by miR-34a.

### miR-34a pull-downs identify new miR-34a targets that regulate cell cycle progression

**Figure 7:**
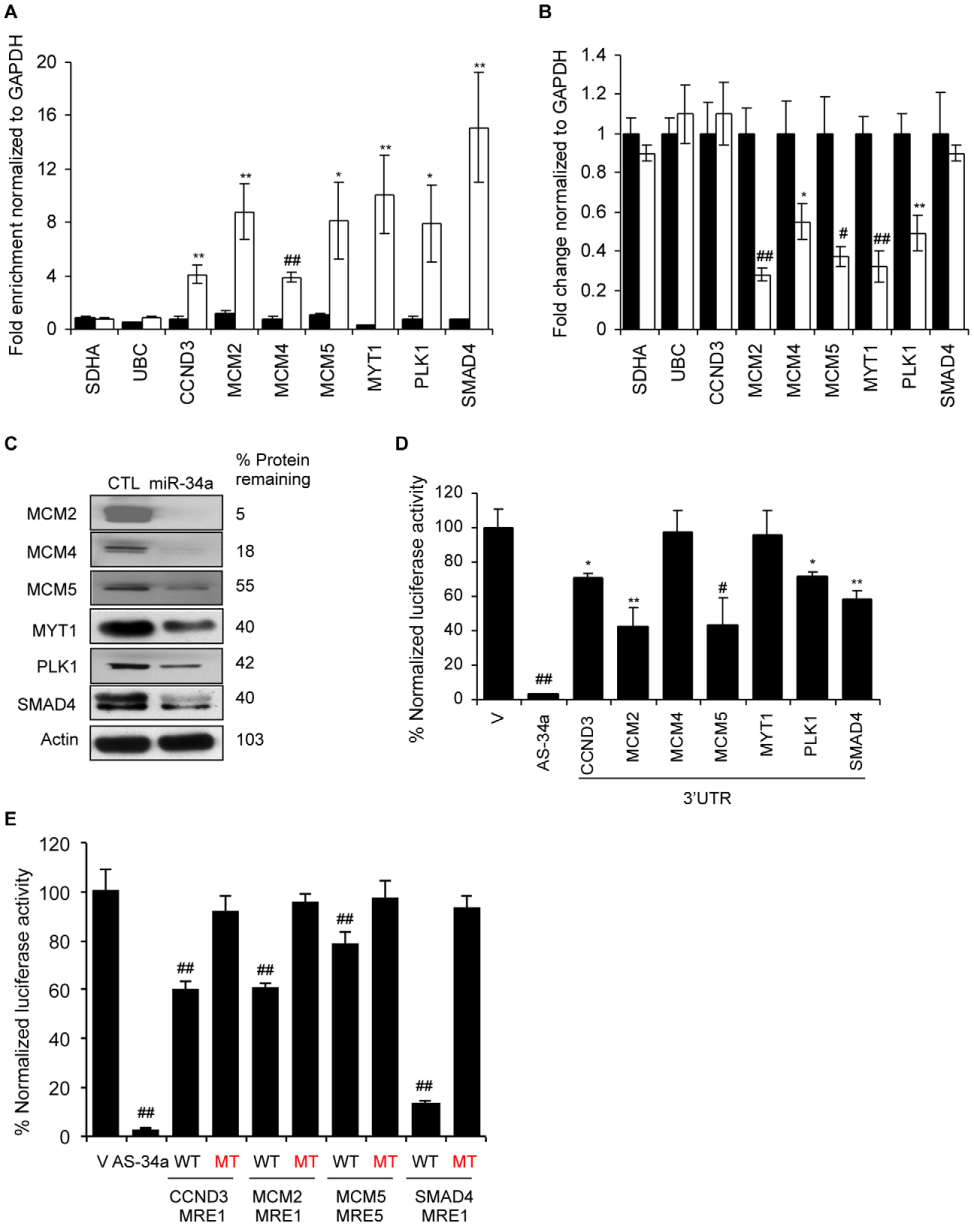
miR-34a pull-downs identify novel miR-34a targets involved in cell cycle progression. (A) Bi-miR-34a pull-down captures transcripts of cell cycle genes. Enrichment of candidate mRNAs in HCT116 cells transfected with Bi-cel-miR-67 miRNA (CTL, black) or Bi-miR-34a (white) for 24 hr was assessed by qRT-PCR analysis normalized to *GAPDH*. *SDHA* and *UBC* mRNAs are housekeeping genes not enriched in the pull-downs. (B) miR-34a over-expression significantly suppresses mRNA levels of 5 of 7 cell cycle genes tested. mRNA expression was analyzed by qRT-PCR relative to *GAPDH* performed on total RNA harvested from HCT116 cells transfected with CTL miRNA (black) or miR-34a (white) mimics for 48 hr. Two candidate target genes (*CCND3* and *SMAD4*) and the housekeeping genes *SDHA* and *UBC* are not significantly altered. (C) Protein levels of 6 of 6 cell cycle genes examined decrease with miR-34a over-expression. HCT116 cells were transfected with CTL miRNA or miR-34a mimics for 48 hr before immunoblot. β-Actin is a loading control. (D) miR-34a represses the 3′UTR of *CCND3*, *MCM2*, *MCM5*, *PLK1* and *SMAD4*, but not *MCM4* and *MYT1*. HeLa cells were cotransfected for 48 hr with CTL miRNA or miR-34a mimics and psiCHECK-2 empty vector (V) or psiCHECK-2 containing the 3′UTR of each gene in the *Renilla luciferase* 3′UTR. The positive control reporter contained a perfectly complementary sequence to miR-34a (AS-34a). Relative luciferase activity in miR-34a-transfected cells is normalized to CTL miRNA-transfected cells. (E) Dual luciferase reporter plasmids bearing wild-type (WT) MREs from *CCND3*, *MCM2*, *MCM5* and *SMAD4* are significantly repressed by co-transfection with miR-34a in HeLa cells. Mutation of the seed region of each MRE (MT) rescues reporter expression. MRE sequences are provided in Figure S5. *, p<0.05, #, p<0.01, **, p<0.005, ##, p<0.001.

Ectopic expression of miR-34a reduces expression of multiple direct target genes whose products facilitate the G_1_/S transition (*CDK4*, *CDK6*, *CCND1*, *CCNE2* and *E2F3*). The pull-down identified novel genes acting at the G_1_/S transition and genes involved in DNA replication and mitosis. Two cell cycle-regulating genes enriched in the miR-34a pull-down are in the random gene list and were already shown (Figure 2C–2E) to be miR-34a-regulated - *CCNG2*, which is most highly expressed in late S phase, and *MAD2L2*, a component of the mitotic spindle assembly checkpoint complex. To examine whether some of the other putative targets that participate in cell cycle progression are direct miR-34a targets, we focused on genes that were both pulled down and down-regulated by miR-34a in HCT116 cells (Table S2). Fourteen cell cycle-regulating genes (*CDK4*, *CDK6*, *CCNE2*, *E2F2*, *E2F3*, *E2F5*, *HDAC1*, *CDKN2A*, *MCM5*, *PKMYT1*, *PLK1*, *SMAD4*, *MAD2L2* and *CCND3*) met these criteria. Four of these (*CDK4*, *CDK6*, *CCNE2* and *E2F3*) are known miR-34a targets. We experimentally tested 5 of the 9 putative novel targets. These genes were *CCND3*, a cyclin that binds to CDK4 or CDK6 and regulates Rb phosphorylation; *MCM5*, a mini-chromosome maintenance (MCM) protein involved in initiating DNA replication, *MYT1*, a serine/threonine protein kinase that phosphorylates and inactivates CDC2, thereby negatively regulating cell cycle progression at the G_2_/M transition; *PLK1*, a serine/threonine protein kinase required for mitotic spindle maturation; and *SMAD4*, a TGFβ-activated transcription factor that induces G_1_ arrest and apoptosis. To determine whether these miR-34a pull-down genes are *bona fide* miR-34a target genes, we first verified that their transcripts associate with Bi-miR-34a (Figure 7A). After miR-34a over-expression, 3 of the 5 genes (*MCM5*, *PLK1* and *MYT1*) had reduced mRNA by at least 2-fold (Figure 7B) and all 5 had significantly reduced protein (Figure 7C). Two other MCM genes, *MCM2* and *MCM4*, also demonstrated a significant miR-34a-dependent reduction in mRNA, and their protein levels became undetectable in miR-34a-transfected cells.

To investigate whether these 5 genes are directly regulated, we measured changes in luciferase activity in HeLa cells after miR-34a co-transfection with reporters containing their 3′UTRs. The 3′UTRs of 4 of 5 of these genes (*CCND3*, *MCM5*, *PLK1* and *SMAD4*) were significantly repressed 30–60% by miR-34a (Figure 7D). The 3′UTR of *MYT1*, which bound to Bi-miR-34a and was down-regulated by miR-34a over-expression (Figure 7A, 7B), was not regulated by miR-34a. *MYT1* expression could be regulated by MREs outside the 3′UTR or indirectly. PITA and TargetScan were used to identify miR-34a MREs in the 3′UTRs of *CCND3*, *SMAD4*, *MCM5*, and *PLK1* (Figure 7E, Figure S5). *CCND3* MRE1, *SMAD4* MRE1 and *MCM5* MRE5 were significantly suppressed by miR-34a (Figure 7E, Figure S5). The *CCND3* and *SMAD4* MREs were predicted by TargetScan, while *MCM5* MRE5 contains a miR-34a hexamer seed match. Mutations that disrupt base pairing with miR-34a rescued luciferase expression, further confirming that these genes are direct miR-34a targets. Because the enrichment ratios for *MCM2* and *MCM4* in the pull-down (∼2.3) were close to our cut-off, we also evaluated whether *MCM2* and *MCM4* might be direct targets. *MCM2* is a direct target as verified by mRNA enrichment in the pull-down, decrease in mRNA and protein following miR-34 over-expression, miR-34a regulation of its 3′UTR by luciferase activity and MRE identification (Figure 7A–7E). However, the *MCM4* 3′UTR was not active in luciferase assays. Collectively, these findings suggest that miR-34a acts as a master regulator of cell proliferation, directly suppressing many key genes that control cell cycle progression.

## Discussion

Despite improvements in bioinformatic and experimental tools, distinguishing the direct targets of a miRNA from indirectly regulated genes remains challenging [14]. Here we describe a simple biochemical method to isolate candidate miRNA targets by streptavidin pull-down of mRNAs that associate with a transfected Bi-miRNA, and apply it to study miR-34a. Comparison of the set of mRNAs that directly associate with the Bi-miRNA with mRNAs down-regulated by miRNA over-expression makes it possible to distinguish the direct and indirect effects of a miRNA. Candidates identified by Bi-miR-34a pull-down have properties of validated miRNA targets: they are enriched for sequences complementary to the miR-34a seed and tend to decrease in expression with miR-34a over-expression. Genes that both decrease in mRNA abundance after over-expression and are isolated by Bi-miR-34a pull-down are further enriched for seed matches, indicating that either they are more likely true miR-34a targets or that a perfect seed match might enhance target mRNA degradation.

In our analysis we defined candidate direct targets using an arbitrary enrichment ratio cut-off of 1 SD, which corresponded to an enrichment of ≥2.5-fold for HCT116 cells and ≥3.3-fold for K562 cells. As the enrichment ratio cut-off was increased, mRNA suppression after ectopic miR-34a expression increased in tandem (Figure 2D). A more stringent cut-off would reduce the already low false positive rate, but also reduce the sensitivity to detect direct targets (Figure 2B). With this cut-off, we identify 71% of the known miR-34a targets expressed in HCT116 cells as “hits”, but only 48% of the known expressed targets in K562 cells. If we had also chosen a 2.5-fold cut-off for K562 cells, our sensitivity for picking targets would have increased to 55%, while a 2-fold cut-off would have increased it to 69%. Since 10 of 11 genes in the random list of genes enriched by ≥2.5 fold by Bi-miR-34a pull-downs in both cells have 3′UTRs regulated directly by miR-34a by luciferase assay, a lower cut-off for the enrichment ratio might have increased sensitivity without an unacceptable false discovery rate. Some bona fide target genes are only enriched in the pull-down by ∼2-fold; one of the novel genes we validated by identifying its MRE (*MCM2*) was only enriched by 2.3-fold in the pull-down of both cell lines. The low false positive rate of target identification demonstrated with the random gene list was also supported by the high degree of experimental validation of the growth factor signaling and cell cycle regulatory genes we chose to examine experimentally (Table S2). In all, we provided experimental evidence for 14 novel direct targets of miR-34a and identified 14 miR-34a MREs, of which 11 had a perfect hexamer seed match and the 3 others had perfect matches if G∶U wobbles were allowed. Thus, the majority of genes we identified as regulated by miR-34a contain canonical 3′UTR MREs with good seed pairing. In the setting of over-expression by transfection, protein levels of all 11 genes we analyzed by immunoblot declined substantially. The few target genes that we tested for which we did not find miR-34a regulation of the 3′UTR might be false positives or might be direct targets, regulated by sequences in the 5′UTR or CDS. In fact we found enrichment for hexamer seed matches in these regions in the mRNAs pulled down with miR-34a, consistent with MRE properties in recent cross-linking-RISC immunoprecipitation experiments [23], [25].

Known targets may not have been identified by the pull-down for a variety of reasons. First, not all of the targets in the literature may be correctly assigned. Second, some known targets, such as CD44, are only modestly regulated by miR-34a [40]. The ratio that defines a “hit” is arbitrary. We set a relatively high threshold for identifying “hits” to maximize the specificity of the method (especially given the large numbers of enriched mRNAs in the pull-down), which came at the cost of sensitivity. Some known targets, which we did not designate hits with our 1 S.D. threshold of the enrichment ratio (which corresponded to >3.3 in K562 cells) had enrichment ratios of 2.5–3.2 in K562 cells. Other bona fide targets may have low, but detectable expression levels, and could have been missed due to the low sensitivity and inter-assay variability of microarray experiments. In addition to cellular variation in endogenous miRNA expression and RISC abundance, other context-dependent biological factors, such as target site accessibility, might vary due to the expression of RNA binding proteins, which could influence the efficiency of miRNA target site binding and the mechanism of targeting [51], [52]. Cell-type specific expression of other MRE-containing genes that compete for miRNA binding could also influence the pull-down enrichment ratio [53]. Finally, some missed targets are likely to be false negatives.

Normalizing the pulled down mRNAs to their abundance in the input cellular mRNA was critical to eliminate from consideration highly abundant housekeeping mRNAs. Our pull-down method modified a previously developed protocol [41], [42], which did not normalize the pull-down mRNAs to the input RNA. Many of the “hits” pulled down with Bi-miR-10a included ribosomal mRNAs, which may represent background binding of very abundant transcripts. Moreover, the miR-10a “hits” were not enriched for mRNAs containing miR-10a 3′UTR seed matches and were not down-regulated by miR-10a over-expression. In other work to be presented elsewhere, the pull-down method was used to identify genome-wide targets of miR-200c and miR-21. Importantly, the miR-200c and miR-21 pulled down mRNAs are also enriched for known targets and for 3′UTR seed sequences.

An advantage to the Bi-miRNA pull-down method described here is its simplicity. In contrast to mRNA expression-based target identification methods, Bi-miRNA pull-downs should identify only direct targets, excluding genes whose expression is indirectly modulated by changes in miRNA expression. Because the degree of mRNA suppression mediated by miRNAs is often small relative to changes in protein, methods that rely on changes in mRNA expression in response to manipulation of miRNA levels will necessarily miss some direct targets. Although the enrichment ratio takes into account a reduction in target gene mRNA in its denominator, the pull-down should not only identify target genes whose mRNA levels decline, but also those that are regulated primarily by inhibiting translation. Unlike approaches based on Ago pull-downs, the Bi-miRNA pull-down identifies the mRNAs directly associated with a specific miRNA, simplifying analysis of biological processes regulated by the miRNA.

The method described here without cross-linking does not directly identify MREs. The streptavidin pull-down method might, however, readily be modified to include cross-linking, RNase digestion of unbound mRNA segments and sequencing, similar to the HITS-CLIP protocol [23], [24], to capture not only direct targets, but also identify MREs of an individual Bi-miRNA. Isolating RNAs associated with an individual miRNA rather than all RISC-associated RNAs in cells over-expressing the miRNA of interest might be a more direct way to define specific target sequences. Future bioinformatic studies of Bi-miRNA pull-down datasets could be used to better define in an unbiased manner the sequence features that dictate miRNA targeting, and could reveal non-canonical modes of targeting, such as those that contain only partial seed complementarity [17] or pairing to the central region of the miRNA [18] or that lie outside the 3′UTR. Indeed, in this work, we enriched for mRNAs with 5′UTR and CDS seed matches, indicating that some direct miR-34a targets may be regulated outside of their 3′UTR.

Only 29% of the 2416 enriched genes in the HCT116 pull-down had down-regulated mRNA levels by mRNA microarray analysis after over-expressing miR-34a for one day, while 10 of 11 randomly chosen genes in the pull-down had significantly decreased mRNA by qRT-PCR analyzed 72 hr after transfection. Thus although miRNAs may commonly lead to mRNA degradation, the degree of mRNA down-regulation of most genes is slight if cells are harvested within a day of transfection. mRNA microarrays may be too noisy to detect subtle changes in expression, unless the analysis is performed on many replicates. Our data also suggest that the kinetics of mRNA degradation may be slow. The early 24 hr time point used for the assay may have fortuitously enhanced our ability to capture miRNA-bound transcripts before too many had been degraded. Indirect effects of the miRNA are also likely to increase over time. The set of genes enriched in the miR-34a pull-down of both HCT116 and K562 cells contains 76 transcription factors or co-factors, whose suppression would reduce many mRNAs.

One important corollary of our results is that miR-34a likely directly regulates hundreds of genes. However, further experimental work is needed to assess how many of the hundreds to thousands of genes whose mRNAs associated with ectopic miR-34a are actually directly regulated by endogenous miR-34a. Possibly only a minority of potential targets is indeed directly regulated in an individual cell at any time. Based on our analysis (Figure 2D), the genes whose transcripts are most enriched in the pull-down may be the most significant targets in a given context. Additional experiments are needed to probe the functional consequences of miR-34a regulation of the genes we identified as targets. The directly regulated genes might vary considerably from cell type to cell type or even in the same cell lineage depending on differentiation state or environmental conditions. For this study we focused on the shared targets identified in two very different types of cells, rather than the ones that were unique to each cell-type. The pull-down method could be used in the future to compare miRNA target genes in different cellular contexts. Notably, the effect of miR-34a on cell signaling differed in the cancer cells we examined. Basal phosphorylation of AKT and ERK was reduced by miR-34a over-expression in HCT116 and HeLa cells (Figure 5), but not in A549 cells (Figure S4). Constitutively active RAS in A549 cells may override the effect of miR-34a in that context. Our results suggest that a dense network of genes that participate in common pathways, sometimes with opposing functions, is capable of being regulated by one miRNA. Although we observed a clear effect of genetic loss of miR-34a on the ability to cells to survive growth factor withdrawal, we did not see reduced expression in miR-34a^−/−^ compared to wild-type cells of some of the key miR-34a target genes we identified. Since growth factor signaling is so central to cell survival and proliferation, the permanent loss of miR-34a expression likely led to myriad compensatory changes. This seeming paradox supports the conclusions of our study – namely that a single miRNA may exert its biological effect by regulating expression of hundreds of genes. The capacity of miR-34a to potentially regulate so many genes that affect growth factor signaling may enable it to exert an effect in diverse contexts.

The numbers of genes that are actually regulated by miR-34a in any setting will likely depend on how strongly miR-34a is expressed. In our pull-down, we greatly over-expressed miR-34a. However, the level of over-expression throughout this study was not greater than endogenous miR-34a expression in some physiological settings, i.e. in K562 cells stimulated with phorbol ester where miR-34a increases 1000-fold [9]. There may be a target gene hierarchy – some genes regulated by low levels of miR-34a, others regulated only by high levels.

The dense network of cell signaling genes captured in the pull-downs suggests that an important function of miR-34a is to regulate the proliferative and activation responses to extracellular growth factors. Despite its function in regulating growth factor signaling and cell proliferation, we did not find a significant variation in miR-34a expression after serum starvation or when cells were synchronized in different phases of the cell cycle (data not shown). In this study we experimentally verified as direct miR-34a targets 5 growth factor signaling genes (*ARAF*, *AXL*, *MEK1*, *MET* and *PIK3R2*). miR-34a was previously shown to inhibit the G_1_/S transition [3], [8]. Here we identified 7 novel cell cycle-regulating direct targets that included genes also required for DNA replication and mitosis. The ultimate anti-proliferative effect of miR-34a integrates both direct consequences of suppressing expression of genes required for progression through the G1/S transition and at other steps of the cell cycle as well as indirect anti-proliferative effects from repressing the growth factor signaling pathways that activate cell cycle progression. Consistent with our genome-wide target gene analysis, miR-34a expression resets the basal state of ERK and AKT phosphorylation in several cell lines, rendering cells less responsive to growth factor signaling (Figure 5). This was shown both by miR-34a overexpression as well as by genetic deletion. miR-34a may reduce cellular sensitivity to growth factor signaling by suppressing many genes in multiple signal transduction pathways. miR-34a candidate targets include genes that are universally involved in transmitting growth factor activation signals as well as some that participate in specific pathways. The particular signaling genes that are suppressed in a given cell line will likely vary from cell to cell, depending on the growth factors to which the cell responds. These types of differences likely contribute to the incomplete overlap between the enriched pathways captured in the two hematopoietic and colon cancer cell lines examined here.

## Materials and Methods

### Cell lines

HCT116, K562, A549 and HeLa cells were from ATCC. miR-34a^+/+^ and miR-34a^−/−^ MEFs were generated from E14.5 littermate embryos. A full description of the mice will be published elsewhere. MEFs were transformed by infecting the cells with retroviruses encoding H-RAS-V12 and E1A and by selection with puromycin (1 µg/ml) and hygromycin (50 µg/ml). The plasmids for expression of H-RAS-V12 (plasmid 9051) and E1A (plasmid 18748) were obtained from Addgene. The VSV-G pseudotyped viruses were produced in 293T cells using the standard protocol. MEFs, HCT116, A549 and HeLa cells were grown in DMEM with 10% fetal bovine serum and supplemented with penicillin, streptomycin, HEPES, L-glutamine and β-mercaptoethanol, K562 cells were grown in RPMI containing 10% fetal bovine serum and the same supplements.

### Transfection of miRNA mimics and plasmid DNA

For most experiments, 2×10^6^ HCT116 or K562 cells were transfected with 200 pmol hsa-miR-34a or cel-miR-67 miRNA mimics (Dharmacon), using Amaxa nucleofection according to the manufacturer's protocol. Biotin was attached to the 3′-end of the active strand. HeLa and A549 cells were transfected with Lipofectamine 2000 and miRNA mimics at a final concentration of 50 nM (Invitrogen). To study the association of Bi-miRNAs with HA-Ago1 or HA-Ago2, pIRESNeo (Clontech) or pIRESNeo-HA-Ago1 or pIRESNeo-HA-Ago2 (Addgene) plasmids were co-transfected in six-well plates (2 µg/well, 1×10^6^ cells/well) with 200 pmol Bi-miR-34a or Bi-cel-miR-67 using Amaxa as per the manufacturer's instructions.

### RNA isolation and quantitative RT–PCR

Total RNA was isolated using Trizol reagent (Invitrogen), treated with DNase I (Ambion) and reverse transcribed using random hexamers and superscript III reverse transcriptase (Invitrogen). qRT-PCR was performed in triplicate samples using SYBR Green FastMix (Quanta) on a BioRad CFX96. mRNA levels were normalized to housekeeping genes *GAPDH*, *UBC* or *SDHA*. miRNA was quantified in triplicate using the TaqMan MicroRNA Assay (Applied Biosystems) as per the manufacturer's instructions and normalized to U6. Primer sequences are listed in Table S3.

### Immunoblot

Whole cell lysates from transfected K562 or HCT116 cells were prepared using RIPA buffer. Proteins were analyzed by SDS-PAGE, transferred to nitrocellulose membranes and probed with the following antibodies: AXL [4566], ARAF [4432], MEK1 [9124], CDK4 [2906], MCM2 [3619], PKMYT1 [4282], PLK1 [4513], SMAD4 [9515], FOXP1 [2005], RBBP4 [4633], AKT [9272], pAKT ser-473 [4051], ERK [4370], pERK [9107] from Cell Signaling; MET [sc-161], MCM5 [sc-165995], E2F1 [sc-251], E2F3 [sc-879], CHEK1 [sc-8408] from Santa Cruz; ACSM3 [SAB1400253], MAD2L2 [SAB1400387], AGBL5 [AV53752], CCNG2 [AV03032], PSMD5 [WH0005711M1] from Sigma; MCM4 [06-1296] from Millipore; and PI3KR [610045], BD Biosciences. Western Blots were quantified by densitometry.

### Biotin pull-down

HCT116 or K562 cells (1×10^6^) were transfected in triplicate with Bi-miR-34a or Bi-cel-miR-67 (Dharmacon) as described above and then cultured in six-well plates. Twenty-four hours later, the cells from 3 wells were pelleted at 500×g. After washing twice with PBS, cell pellets were resuspended in 0.7 ml lysis buffer (20 mM Tris (pH 7.5), 100 mM KCl, 5 mM MgCl_2_, 0.3% NP-40, 50 U of RNase OUT (Invitrogen), complete mini-protease inhibitor cocktail (Roche Applied Science)), and incubated on ice for 5 min. The cytoplasmic lysate was isolated by centrifugation at 10,000×g for 10 min. Streptavidin-coated magnetic beads (Invitrogen) were blocked for 2 hr at 4°C in lysis buffer containing 1 mg/ml yeast tRNA and 1 mg/ml BSA (Ambion) and washed twice with 1 ml lysis buffer. Cytoplasmic lysate was added to the beads and incubated for 4 h at 4°C before the beads were washed five times with 1 ml lysis buffer. RNA bound to the beads (pull-down RNA) or from 10% of the extract (input RNA), was isolated using Trizol LS reagent (Invitrogen). The level of mRNA in the Bi-miR-34a or Bi-cel-miR-67 control pull-down was quantified by qRT-PCR or mRNA microarray. For qRT-PCR, mRNA levels were normalized to a housekeeping gene (*GAPDH*, *SDHA* or *UBC*). The enrichment ratio of the control-normalized pull-down RNA to the control-normalized input levels was then calculated.

### Microarray analysis

Total RNA (independently in two experiments) was amplified, labeled and hybridized to Affymetrix U133 plus 2.0 mRNA microarrays. The quality of the RNA was assessed before performing the microarray and the quality of the microarray data was assessed using affyPLM and Affy software. The replicate data sets for the 4 sets of samples (pull-down and input for miR-34a and cel-miR-67) were compared using an unsupervised hierarchical clustering algorithm, which verified the similarity of the duplicates. The microarray data were normalized using RMA [7] to reduce interarray variation. The enrichment ratio {Bi-miR-34a PD/Bi-cel-miR-67 PD}/{Bi-miR-34a input/Bi-cel-miR-67 input} was calculated for each probe. For genes represented by multiple probes, the mean ratio for all the probes was calculated. Genes for which none of the probe hybridization signals exceeded the background were considered not expressed and were disregarded in the analysis. For informatic analysis of the PD data, genes whose enrichment ratio were ≥1 SD above background based on a log-normal distribution were considered “hits”.

### Gene down-regulation after miR-34a over-expression

HCT116 or K562 cells were transfected in independent duplicate experiments as above with unbiotinylated miR-34a or cel-miR-67 (Dharmacon) and total RNA was harvested 24 hr later and analyzed as above by gene expression microarrays. After normalization, fold changes for each probe were calculated as the ratio of input RNA from miR-34a-transfected cells to the ratio of input RNA from cel-miR-67-transfected cells. Genes were considered down-regulated if the ratio decreased by at least 20%, which corresponded to ∼1 SD. To test the expression levels of putative target sets, each gene list was plotted in a cumulative distribution function (CDF) plot, and the Kolmogorov-Smirnov [KS] test was used for statistical comparisons between gene sets.

### Analysis of miR-34a target genes by target prediction algorithms

To determine whether a gene was also a predicted target of miR-34a, the presence of miR-34a binding sites was analyzed using TargetScan 4.2 (http://www.targetscan.org/) [39], [54], [55] or PITA (http://132.77.150.113/pubs/mir07/mir07_prediction.html) [50].

### Hexamer analysis

The mature hsa-miR-34a sequence was obtained from miRBase (http://mirbase.org/). All RefSeq human mRNA sequences were downloaded from NCBI in July 2009 (http://ftp.ncbi.nih.gov/). mRNAs were indexed by Entrez Gene ID; in cases where multiple sequences matched a gene ID, the sequence with the longest 3′UTR was selected. For each test gene list and miR-34a hexamer, the miR-34a hexamer frequency (hexamer matches per kb of sequence) was calculated. The frequency of hexamer matches for all genes on the microarray (the background set) was also determined. Gene IDs with no corresponding sequence in the database were excluded from analysis. Monte Carlo simulations of equally sized random gene sets (without replacement) were used to generate an empirical 2-tailed p-value for each gene set/hexamer combination. When p<1E-4, the p-value was calculated from curve fitting relative to the random background distribution.

### Pathway enrichment analysis and network visualization

For each of the lists of down-regulated and pull-down-enriched genes, the p-value of over-representation in a suite of canonical pathways (KEGG [56] and Wikipathways [57]) was determined using the hypergeometric distribution. A visualization of the relationship between the enriched pathways (p<0.001) based on the number of overlapping genes was rendered using Cytoscape [58]. The network of gene-gene interactions underlying these relationships was constructed based on interactions supplied by MetaCore (GeneGo Inc). Physical, predicted and genetic interactions were used to connect the down-regulated and pull-down enriched genes within the significant signaling, cell cycle or DNA repair pathways. Signaling pathway genes with no connection to any other node were removed and the network was arranged according to predicted sub-cellular localization.

### Luciferase assay

HeLa cells were cotransfected in 24 well plates using Lipofectamine 2000 (Invitrogen) with 50 nM miR-34a mimic or control miRNA mimic and 50 ng of psiCHECK2 (Promega) vector containing the MRE or 3′UTR of indicated genes cloned into the multiple cloning site of *Renilla* luciferase. After 48 hr of transfection (unless otherwise indicated) luciferase activities were measured using the Dual Luciferase Assay System (Promega) and Top count NXT microplate reader (Perkin Elmer) per manufacturer's instructions. All experiments were performed at least in triplicate. Results were normalized to those obtained in cells transfected with an empty vector. For some experiments, a perfectly complementary antisense sequence to the active strand of miR-34a was inserted into the multiple cloning site for use as a positive control. Data were normalized to *Firefly* luciferase and results from 3 independent experiments were compared. Sequence of primers used for cloning 3′UTRs for miR-34a target genes are listed in Table S4. MREs sequences were cloned into psiCHECK-2 by annealing complementary oligomers matching each MRE sequence (Figures S4, S5) with overhanging ends complementary to the XhoI and NotI sites of psiCHECK-2.

### Cell growth experiments

HCT116, HeLa and A549 cells were transfected as described above. One day after transfection, cells were placed in serum-free medium or medium containing 10% fetal calf serum. 48 hours after the medium was changed, total cell numbers were counted. MEFs were plated at a density of 2.5×10^5^ or 5×10^5^ cells per well of a 6-well plate. The medium was changed to vary serum concentration 24 hr after plating. The MEFs were harvested 24 hr later and counted using Trypan blue staining or stained in PBS+0.4% BSA with annexinV-APC (Invitrogen) at a 1∶30 dilution, then washed once and stained with propidium iodide (4 µg/ml) (Sigma-Aldrich).

## Supporting Information

Figure S1 (A) HCT116 cells were transfected with Bi-miR-34a or Bi-cel-miR-67 (CTL) and after 24 hr, abundance of known miR-34a target mRNAs (*CDK4*, *CDK6* and *MYB*) was measured by qRT-PCR analysis of pull-down RNA. *CDK4*, *CDK6* and *MYB* and not *UBC* (a housekeeping mRNA) were significantly enriched in the Bi-miR-34a pull-downs (white) and not the control pull-down (black). (B) K562 cells were transfected with Bi-miR-34a (white) or Bi-CTL (black), and RNA isolated from the streptavidin pull-down was analyzed by qRT-PCR for miR-34a and miR-24 (a control miRNA) after normalization to *U6*. miR-34a was ∼50-fold higher in miR-34a pull-down as compared to control pull-down. miR-24 was not enriched and its levels were similar in each pull-down. (C) Addition of Bi-miR-34a (white) or Bi-CTL (black) to cytoplasmic extracts prepared from untransfected K562 cells does not enrich for known miR-34a target mRNAs, suggesting that the specific association of these mRNAs with Bi-miR-34a occurs in live cells and not post-lysis. Data in (B) are from 3 independent experiments and in (A) and (C) are from duplicate experiments.
(TIF)

Figure S2 Sequence characteristics of Bi-miR-34a pull-down targets. (A) Enrichment of hexamers matching each position of the mature miR-34a sequence in the HCT116 and K562 pull-down (red), down-regulated genes (blue), and genes down-regulated by miR-34a and pulled-down (yellow). Genes both enriched by Bi-miR-34a pull-down and down-regulated by miR-34a are the most enriched for miR-34a seed matches (B) Hexamer enrichment analysis for genes enriched in both HCT116 and K562 Bi-miR-34a pull-downs. Bi-miR-34a pull-down enriched for sequences matching two miRNA regions: the seed (positions 1–8) and a possible 3′ compensatory region (positions 13–19). Bi-miR-34a pull-down mRNAs are also enriched for CDS and 5′UTR matches to these sequences (*p≤0.0001).
(TIF)

Figure S3 Pathway networks representing the significant canonical pathways enriched for TargetScan conserved (A) and TargetScan non-conserved (B) target predictions.
(TIF)

Figure S4 miR-34a regulation of growth factor signaling. (A) Western blots of A549 cells transfected with miR-34a or CTL mimics. No reproducible change in pERK or pAKT was observed in these cells. (B) A549 cells were transfected with miR-34a or cel-miR-67 (CTL) mimics, and placed in normal growth medium with 10% serum (+) or growth medium lacking serum (−). Cells transfected with miR-34a did not proliferate in response to serum. Candidate miR-34a microRNA recognition elements (MRE) in the 3′UTR of *AXL*, *ARAF*, *MEK1*, *MET* and *PIK3R2* mRNAs predicted by PITA (see Materials and Methods). Numbers in parenthesis represent the location of the MRE in the 3′UTR. Wild-type MREs in (C) were repressed by miR-34a (see Figure 5F) whereas MREs that were not responsive to miR-34a are shown in (D). Point mutations that disrupt the base-pairing with miR-34a are shown in red in the mutant MREs.
(TIF)

Figure S5 Candidate miR-34a microRNA recognition elements (MRE) in the 3′UTR of *CCND3*, *MCM2*, *MCM5*, *PLK1* and *SMAD4* mRNAs predicted by PITA or TargetScan (see Materials and Methods). Numbers in parenthesis represent the location of the MRE in the 3′UTR (*PLK1* MRE2 spans the stop codon of *PLK1*). Wild-type MREs in (A) were repressed by miR-34a (see Figure 6E), whereas MREs that were not responsive to miR-34a are shown in (B). Point mutations that disrupt the base-pairing with miR-34a are shown in red in the mutant MREs.
(TIF)

Table S1 Genes enriched in Bi-miR-34a pull-downs or down-regulated by miR-34a over-expression in HCT116 and K562 cells.
(XLS)

Table S2 Experimental validation of miR-34a target genes.
(XLS)

Table S3 Sequence of primers used for qRT-PCR.
(XLS)

Table S4 Sequence of primers used for cloning 3′UTR of miR-34a target genes.
(XLS)
