# Supplementary figures and images for: Capture of MicroRNA–Bound mRNAs Identifies the Tumor Suppressor miR-34a as a Regulator of Growth Factor Signaling
Source: PLoS Genet. 2011 Nov 10;7(11):e1002363. doi: 10.1371/journal.pgen.1002363 (PMC3213160; doi:10.1371/journal.pgen.1002363)

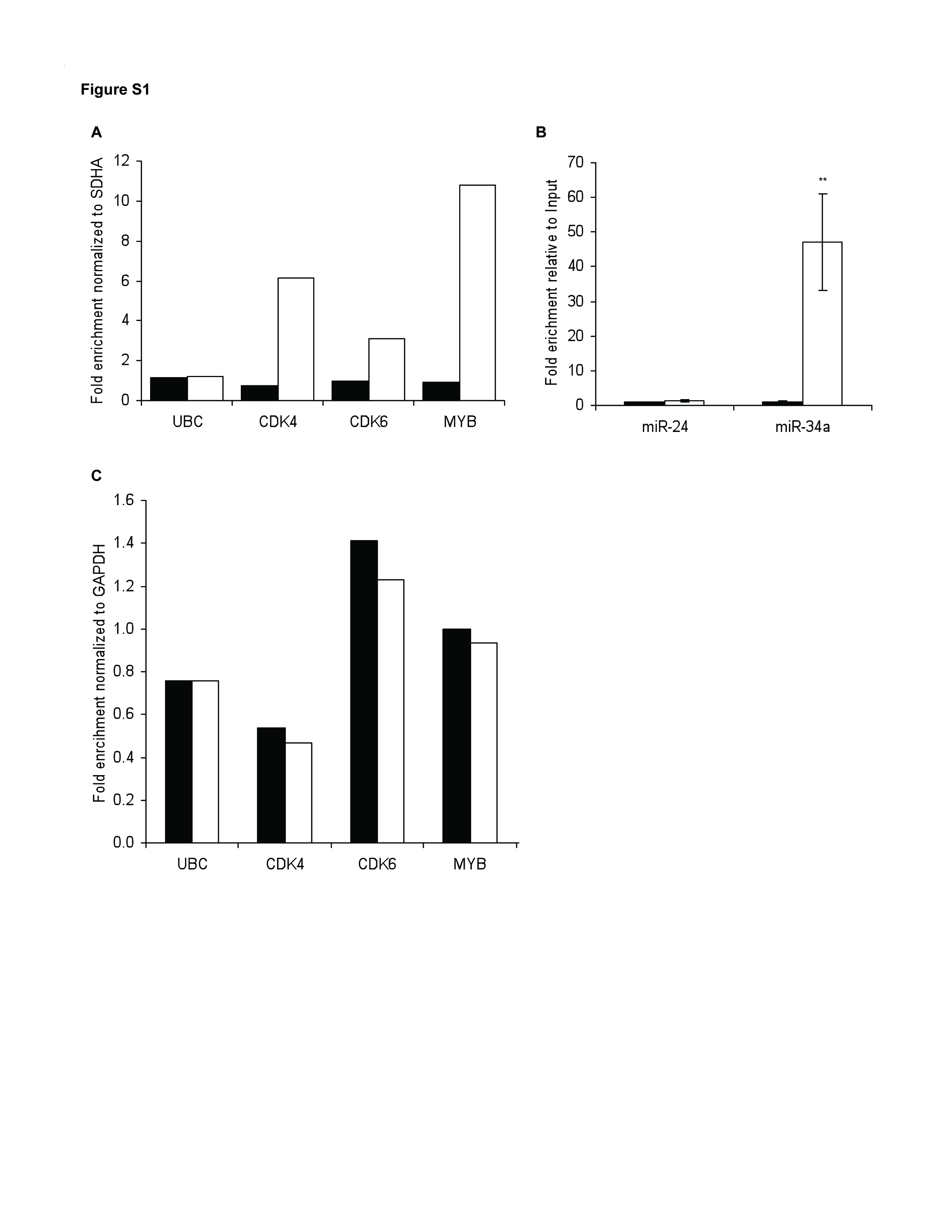

Supplement: Figure S1 — (A) HCT116 cells were transfected with Bi-miR-34a or Bi-cel-miR-67 (CTL) and after 24 hr, abundance of known miR-34a target mRNAs (CDK4, CDK6 and MYB) was measured by qRT-PCR analysis of pull-down RNA. CDK4, CDK6 and MYB and not UBC (a housekeeping mRNA) were significantly enriched in the Bi-miR-34a pull-downs (white) and not the control pull-down (black). (B) K562 cells were transfected with Bi-miR-34a (white) or Bi-CTL (black), and RNA isolated from the streptavidin pull-down was analyzed by qRT-PCR for miR-34a and miR-24 (a control miRNA) after normalization to U6. miR-34a was ∼50-fold higher in miR-34a pull-down as compared to control pull-down. miR-24 was not enriched and its levels were similar in each pull-down. (C) Addition of Bi-miR-34a (white) or Bi-CTL (black) to cytoplasmic extracts prepared from untransfected K562 cells does not enrich for known miR-34a target mRNAs, suggesting that the specific association of these mRNAs with Bi-miR-34a occurs in live cells and not post-lysis. Data in (B) are from 3 independent experiments and in (A) and (C) are from duplicate experiments. (TIF) [file pgen.1002363.s001.tif]

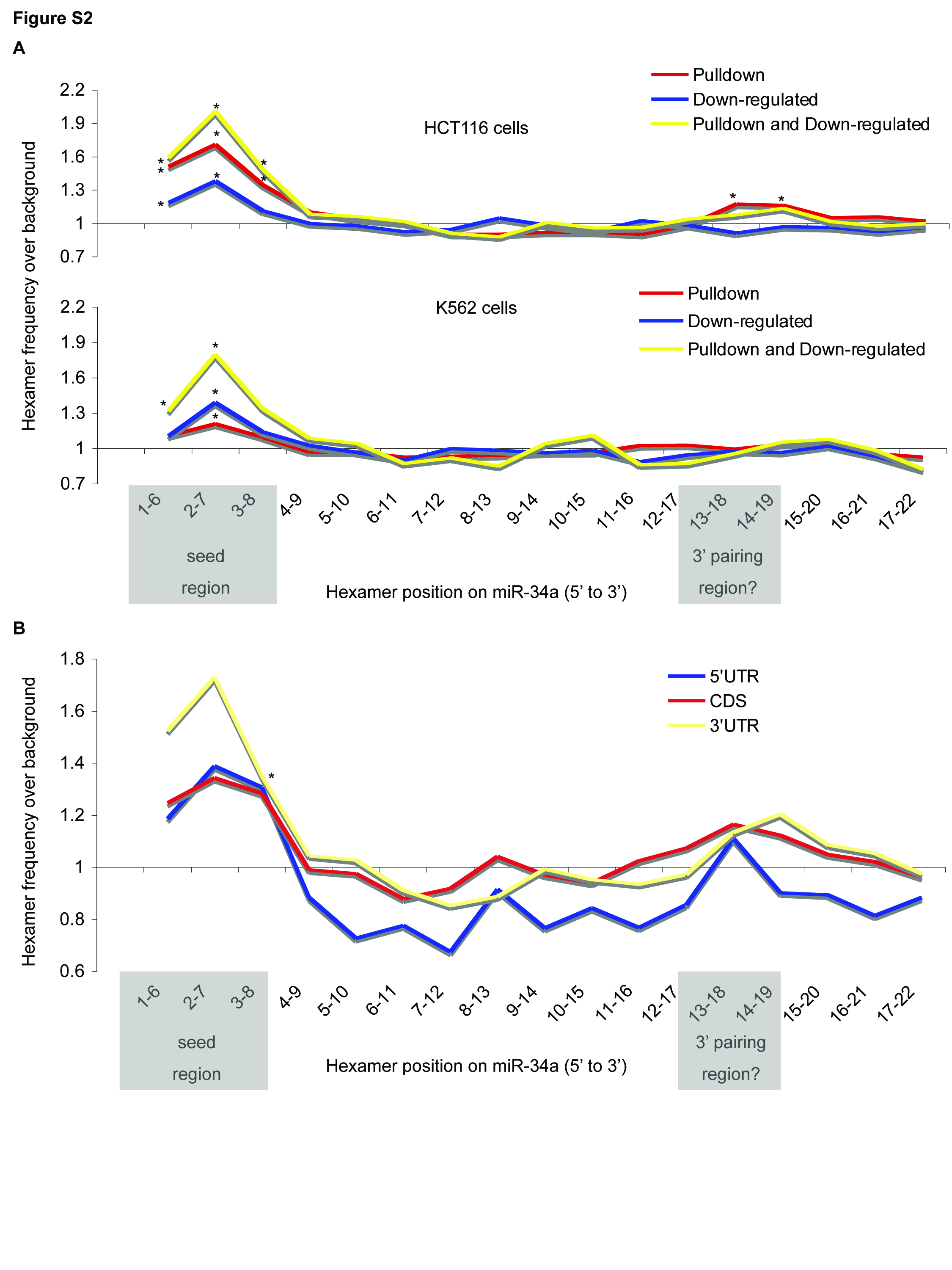

Supplement: Figure S2 — Sequence characteristics of Bi-miR-34a pull-down targets. (A) Enrichment of hexamers matching each position of the mature miR-34a sequence in the HCT116 and K562 pull-down (red), down-regulated genes (blue), and genes down-regulated by miR-34a and pulled-down (yellow). Genes both enriched by Bi-miR-34a pull-down and down-regulated by miR-34a are the most enriched for miR-34a seed matches (B) Hexamer enrichment analysis for genes enriched in both HCT116 and K562 Bi-miR-34a pull-downs. Bi-miR-34a pull-down enriched for sequences matching two miRNA regions: the seed (positions 1–8) and a possible 3′ compensatory region (positions 13–19). Bi-miR-34a pull-down mRNAs are also enriched for CDS and 5′UTR matches to these sequences (*p≤0.0001). (TIF) [file pgen.1002363.s002.tif]

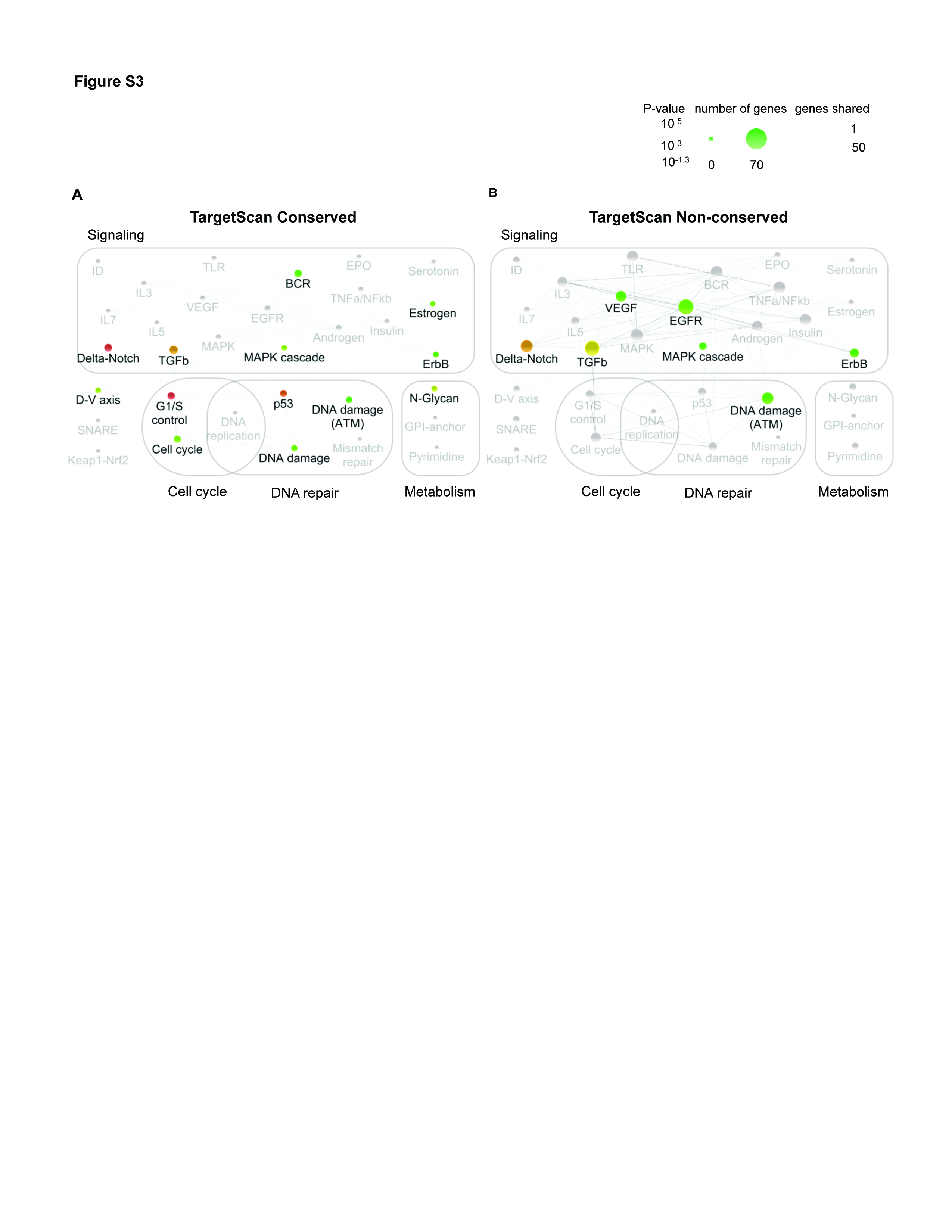

Supplement: Figure S3 — Pathway networks representing the significant canonical pathways enriched for TargetScan conserved (A) and TargetScan non-conserved (B) target predictions. (TIF) [file pgen.1002363.s003.tif]

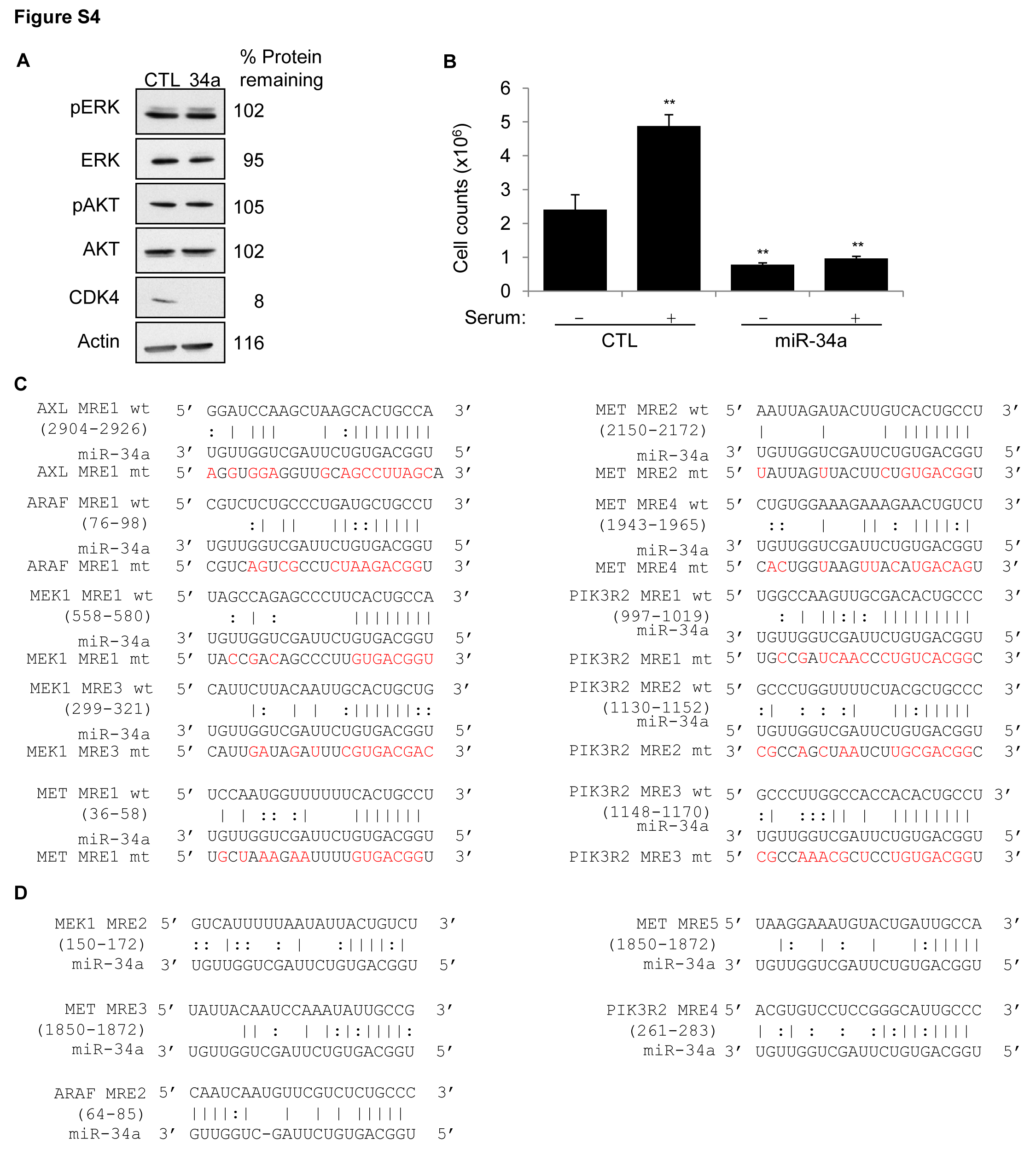

Supplement: Figure S4 — miR-34a regulation of growth factor signaling. (A) Western blots of A549 cells transfected with miR-34a or CTL mimics. No reproducible change in pERK or pAKT was observed in these cells. (B) A549 cells were transfected with miR-34a or cel-miR-67 (CTL) mimics, and placed in normal growth medium with 10% serum (+) or growth medium lacking serum (−). Cells transfected with miR-34a did not proliferate in response to serum. Candidate miR-34a microRNA recognition elements (MRE) in the 3′UTR of AXL, ARAF, MEK1, MET and PIK3R2 mRNAs predicted by PITA (see Materials and Methods). Numbers in parenthesis represent the location of the MRE in the 3′UTR. Wild-type MREs in (C) were repressed by miR-34a (see Figure 5F) whereas MREs that were not responsive to miR-34a are shown in (D). Point mutations that disrupt the base-pairing with miR-34a are shown in red in the mutant MREs. (TIF) [file pgen.1002363.s004.tif]

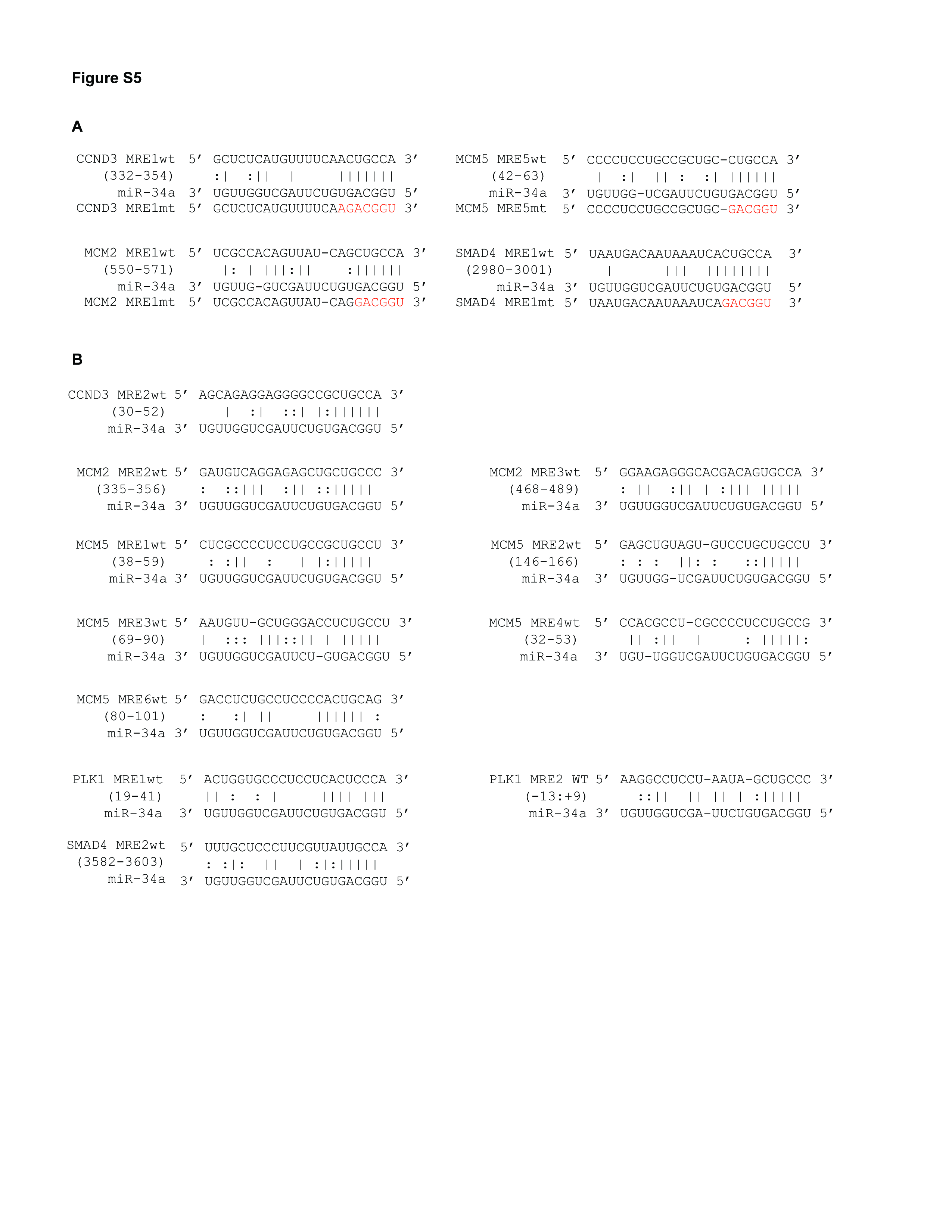

Supplement: Figure S5 — Candidate miR-34a microRNA recognition elements (MRE) in the 3′UTR of CCND3, MCM2, MCM5, PLK1 and SMAD4 mRNAs predicted by PITA or TargetScan (see Materials and Methods). Numbers in parenthesis represent the location of the MRE in the 3′UTR (PLK1 MRE2 spans the stop codon of PLK1). Wild-type MREs in (A) were repressed by miR-34a (see Figure 6E), whereas MREs that were not responsive to miR-34a are shown in (B). Point mutations that disrupt the base-pairing with miR-34a are shown in red in the mutant MREs. (TIF) [file pgen.1002363.s005.tif]
